# Chemically Induced Hypoxia Enhances miRNA Functions in Breast Cancer

**DOI:** 10.3390/cancers12082008

**Published:** 2020-07-22

**Authors:** Emma Gervin, Bonita Shin, Reid Opperman, Mackenzie Cullen, Riley Feser, Sujit Maiti, Mousumi Majumder

**Affiliations:** Department of Biology, Brandon University, 3rd Floor, John R. Brodie Science Centre, 270-18th Street, Brandon, MB R7A6A9, Canada; GERVINEE02@BrandonU.CA (E.G.); SHINBW60@brandonu.ca (B.S.); OPPERMRM72@brandonu.ca (R.O.); CULLENMW01@brandonu.ca (M.C.); FESERRJ36@brandonu.ca (R.F.); MaitiS@BrandonU.CA (S.M.)

**Keywords:** Breast cancer, Hypoxia inducible factor 1-alpha (HIF-1α), MicroRNA (miRNA), miR526b, miR655, Oxidative stress, Migration, Cyclooxygenase-2 (COX-2), Prostaglandin E2 receptor 4 (EP4), PI3K/Akt

## Abstract

In aggressively growing tumors, hypoxia induces HIF-1α expression promoting angiogenesis. Previously, we have shown that overexpression of oncogenic microRNAs (miRNAs, miRs) miR526b/miR655 in poorly metastatic breast cancer cell lines promotes aggressive cancer phenotypes in vitro and in vivo. Additionally, miR526b/miR655 expression is significantly higher in human breast tumors, and high miR526b/miR655 expression is associated with poor prognosis. However, the roles of miR526b/miR655 in hypoxia are unknown. To test the relationship between miR526b/miR655 and hypoxia, we used various in vitro, in silico, and in situ assays. In normoxia, miRNA-high aggressive breast cancer cell lines show higher HIF-1α expression than miRNA-low poorly metastatic breast cancer cell lines. To test direct involvement of miR526b/miR655 in hypoxia, we analyzed miRNA-high cell lines (MCF7-miR526b, MCF7-miR655, MCF7-COX2, and SKBR3-miR526b) compared to controls (MCF7 and SKBR3). CoCl_2_-induced hypoxia in breast cancer further promotes *HIF-1α* mRNA and protein expression while reducing *VHL* expression (a negative HIF-1α regulator), especially in miRNA-high cell lines. Hypoxia enhances oxidative stress, epithelial to mesenchymal transition, cell migration, and vascular mimicry more prominently in MCF7-miR526b/MCF7-miR655 cell lines compared to MCF7 cells. Hypoxia promotes inflammatory and angiogenesis marker (*COX-2*, *EP4*, *NFκB1*, *VEGFA*) expression in all miRNA-high cells. Hypoxia upregulates miR526b/miR655 expression in MCF7 cells, thus observed enhancement of hypoxia-induced functions in MCF7 could be attributed to miR526b/miR655 upregulation. In silico bioinformatics analysis shows miR526b/miR655 regulate *PTEN* (a negative regulator of *HIF-1α*) and *NFκB1* (positive regulator of *COX-2* and *EP4*) expression by downregulation of transcription factors *NR2C2*, *SALL4*, and *ZNF207*. Hypoxia-enhanced functions in miRNA-high cells are inhibited by COX-2 inhibitor (Celecoxib), EP4 antagonist (ONO-AE3-208), and irreversible PI3K/Akt inhibitor (Wortmannin). This establishes that hypoxia enhances miRNA functions following the COX-2/EP4/PI3K/Akt pathways and this pathway can serve as a therapeutic target to abrogate hypoxia and miRNA induced functions in breast cancer. In situ, *HIF-1α* expression is significantly higher in human breast tumors (*n* = 96) compared to non-cancerous control tissues (*n* = 20) and is positively correlated with miR526b/miR655 expression. In stratified tumor samples, *HIF-1α* expression was significantly higher in ER-positive, PR-positive, and HER2-negative breast tumors. Data extracted from the TCGA database also show a strong correlation between *HIF-1α* and miRNA-cluster expression in breast tumors. This study, for the first time, establishes the dynamic roles of miR526b/miR655 in hypoxia.

## 1. Introduction

Breast cancer is the most common form of cancer, as well as the second leading cancer-related death among Canadian women [1]. According to Canadian Cancer Statistics, 1 in 8 Canadian women will develop breast cancer in their lifetime, while 1 in 33 will die from it [1]. Understanding the complexity of the disease is urgently required to find personalized therapy for various kinds (i.e., estrogen receptor (ER)-positive and ER-negative; progesterone receptor (PR)-positive and PR-negative; human epidermal growth factor receptor 2 (HER2)-positive and HER2-negative; and triple-negative (ER-PR-HER2-negative)) of breast cancer, as there is no single target for treating such a complex malignancy. One of the factors that contribute to the complexity of tumor growth, metastasis, and patient survival in breast cancer is the level of hypoxia (oxygen deficiency) within the tumor microenvironment [2]. Due to their rapid proliferation, cancer cells outgrow the available blood supply. This limits the delivery of oxygen and nutrients to the cells, making the center of the aggressively growing tumor largely hypoxic [2]. To counteract hypoxia, cancerous cells secrete growth factors and stimulants that facilitate tumor-associated angiogenesis in the tumor microenvironment to deliver the required oxygen and nutrients to dividing tumor cells [3].

Hypoxia influences multiple signaling pathways in cells, including the hypoxia-inducible factor (HIF), NFκB, ERK, and PI3K/Akt/mTOR pathways, which regulate apoptosis, migration, proliferation, and inflammation in cancer [4,5,6]. HIF-1 is a heterodimer composed of the HIF-1α and HIF-1β subunits. Under normoxia (physiologically normal oxygen levels), both the HIF-1α and -1β subunits are constitutively expressed, but the HIF-1 dimer is not formed as the HIF-1α subunit is degraded in the presence of oxygen [4]. Under normoxic condition, the oxygen-dependent degradation domain of HIF-1α is hydroxylated by the PHD (prolyl hydroxylase domain) enzyme, which further allows the tumor suppressor pVHL (Von Hippel-Lindau) to catalyze the ubiquitin-dependent degradation of the HIF-1α protein [7]. In hypoxic conditions, this hydroxylation does not occur, and pVHL does not catalyze the ubiquitination of the HIF-1α protein, allowing it to avoid degradation. The HIF-1α subunit can then dimerize with the HIF-1β subunit to form HIF-1 [5]. HIF-1 is a transcription factor that binds to promoter regions and regulates the expression of multiple genes, including vascular endothelial growth factors (*VEGFs*) (a pro-angiogenic agent), anaerobic respiration enzymes, glucose metabolism, and regulates microRNA (miRNA, miR) biogenesis and functions [8,9,10]. The net effects of these changes increase the amount of ATP available to the tumor cell, promoting rapid growth.

miRNAs are defined as a group of endogenously-produced, small, non-coding RNAs that can downregulate gene expression of target messenger RNAs (mRNAs) at the post-transcription level by complete or partial complementary base pairing. Dysregulated miRNA expression has been associated with various cancers, including breast cancer [3,11,12,13,14]. Using gene expression and miRNA microarray assays, we have identified that the overexpression of COX-2 in a poorly metastatic MCF7 cells (an ER/PR-positive and HER2-negative breast cancer cell line) upregulates two miRNAs, miR526b and miR655, which have been classified as oncogenic miRNAs in human breast cancer [13,14]. We found that miR526b and miR655 collectively target a total of 13 genes in COX-2 overexpressing MCF7 cells (MCF7-COX2), 12 of which are classified as tumor-suppressor-like genes [15]. The single gene targeted by both miRNAs was identified as cytoplasmic polyadenylation element-binding protein 2 (*CPEB2*). Recently, it was identified that *CPEB2* is a tumor suppressor gene, further validating miR526b and miR655 as oncogenic miRNAs promoting breast cancer by collectively targeting this gene [15]. We have previously shown that in SKBR3, MDA-MB-231, and MCF7-COX2 cell lines COX-2, miR526b, and miR655 were upregulated, while *CPEB2* was downregulated [15]. miR526b is located on a large cluster of miRNAs on chromosome 19 with the chromosomal location 19q13.42, in the gene family miR515 [16,17]. miR655 is located on a large cluster of miRNA on chromosome 14 on the host gene miR381HG in the chromosomal location 14q32.31 and belongs to the miR154 gene family [17,18].

We have also shown that miR526b and miR655 overexpression in ER-positive breast cancer cell line MCF7 and an ER-negative HER2-positive breast cancer cell line SKBR3 promotes epithelial-to-mesenchymal transition (EMT), cell migration, invasion, induction of stem-like cells (SLCs) phenotype, tumor growth, and metastasis in vivo [13,14]. In growing tumors, the core of the mass becomes hypoxic and requires a new means for oxygen delivery. This is achieved through tumor-associated angiogenesis, a phenotype that can be induced by the expression of certain miRNAs [3,19,20]. We have identified that overexpression of miR526b/miR655 in MCF7 cells enhances tumor-associated angiogenesis and lymphangiogenesis by the production of VEGFA and that miRNA cell secretion enhances tube formation in vascular endothelial cells [3]. Cancer cells can also mimic the properties of vascular endothelial cells to induce tumor-associated angiogenesis, known as vascular mimicry [21,22]. We have shown that in human breast tumors, miR526b and miR655 expression is highly correlated with angiogenesis and lymphangiogenesis markers (VEGFA, VEGFC, and VEGFD) [3]. In this article, we investigated the roles of miRNA in promoting angiogenic marker expression and vascular mimicry in hypoxia.

EMT is an important biological process characterized by the progressive loss of cell-to-cell adhesion, alterations in cellular polarity, and actin cytoskeletal rearrangements leading to the formation of filopodia and upregulation of mesenchymal phenotypes and markers [23]. Tumor cells lose intercellular junction proteins such as E-Cadherin (CDH1) and are able to travel through the extracellular matrix, in a process known as cell migration [24]. EMT is necessary for the migration of embryonic cells to establish the development of an embryo, and to complete wound healing in adult tissues. However, EMT in cancer leads to the promotion of aggressive phenotypes, such as migration, invasion, angiogenesis, stem-like phenotypes in cancer cells, and resistance to chemo-radiotherapy [25]. Previously, we have shown that miR526b and miR655 induce EMT in breast cancer, promote tumor cell migration and invasion [13,14], and that miRNA cell secretions enhance the migration of vascular endothelial cells to enhance angiogenesis [3]. However, hypoxia’s influence on miRNA-induced EMT is not clear.

Another known phenotype in hypoxic tumors is the formation of reactive oxygen species (ROS), such as superoxide (SO), which are byproducts of cellular metabolism. Cellular inability to neutralize and eliminate these ROS leads to oxidative stress. Furthermore, increased levels of SO have shown regulation of signaling cascades for cell proliferation and survival [26]. We have shown that a dynamic relationship exists between oxidative stress and miR526b/miR655 expression, where an increase in miRNA leads to an increase in ROS and SO. Likewise, an increase in ROS was shown to significantly increase miR526b and miR655 expression, suggesting that a positive feedback loop relationship between both miRNAs and oxidative stress is present in human breast cancer [27]. We have previously shown that when we treat poorly metastatic breast cancer tumor cell line MCF7 and primary endothelial cell line human umbilical vein endothelial cells (HUVECs) with cell secretions from miR526b and miR655-overexpressing cells, there is an increase in ROS, SO, and oxidative stress marker thioredoxin reductase 1 (TXNRD1) expression. This suggests that miR526b/miR655-high cells’ metabolites induce oxidative stress in the tumor microenvironment. Thus, we wanted to investigate the effect of hypoxia on miRNA-induced oxidative stress in breast cancer cells.

For the first time, with this specific research, we investigate the capability of miR526b, miR655, and hypoxia collaborating to promote aggressive breast cancer phenotypes. First, we show that highly metastatic and miRNA-high cell lines show high expression of *HIF-1α* in normoxia, while poorly metastatic, miRNA-low cell lines show low expression. Next, we used CoCl_2_ to induce hypoxia in ER-positive MCF7, MCF7-miR526b, and MCF7-miR655 cells, as well as HER2-positive SKBR3 and SKBR3-miR526b cells, since CoCl_2_ has long been used as a chemical inducer of hypoxia and has been shown to induce *HIF-1α* expression [28,29]. We further verified the effects of hypoxia enhancing miRNA-induced oxidative stress, cell migration, induction of EMT, expression of hypoxia-linked genes such as *VHL*, *HIF-1α*, and *NFκB1*, and expression of inflammation-associated genes such as *VEGFA*, *COX-2*, and *EP4* in breast cancer cell lines. Here we demonstrated that hypoxia enhances oncogenic miRNA functions in breast cancer, which can be inhibited by COX-2, EP4, and PI3K/Akt signaling pathway inhibitors. In silico bioinformatics analysis further confirms that miRNA functions in hypoxia are regulated by COX-2/EP4/PI3K/Akt pathways and that miRNA has a negative correlation with transcription factors that regulate the expression of *NFκB1* and *PTEN*. In human breast tumors, *HIF-1α* expression is significantly high and we estimated the highest expression in the ER-positive, PR-positive, and HER2-negative breast tumors. Both miR526b and miR655 expression in breast tumors is positively and significantly correlated with *HIF-1α* expression in the set of tumor samples we used in this study and also data extracted from The Cancer Genome Atlas (TCGA) cBioPortal database, which includes data from 16 different breast cancer studies, strongly suggesting that hypoxia and miRNAs collaborate to promote breast cancer progression. This is a novel function of miR526b and miR655 in breast cancer.

## 2. Results

We do not have access to a hypoxic chamber, thus, we used CoCl_2_ to induce hypoxia. CoCl_2_ increases the expression of hypoxic marker HIF-1α and induces hypoxia in MCF7 cells [29]. First, we conducted a dose-response assay of *HIF-1α* expression with various concentrations of CoCl_2_ (Figure S1).

To investigate the interaction between miRNA and hypoxia, we used various breast cancer cell lines with differential levels of miR526b and miR655 expression [13,14]. We used the breast epithelial cell line MCF10A, poorly-metastatic breast cancer cell lines MCF7, T47D (ER-positive, PR-positive, and HER2-negative) and SKBR3 (ER-negative, PR-negative, and HER2-positive); highly metastatic breast cancer cell lines Hs578T, MDA-MB-231 (ER-negative, PR-negative, and HER2-negative), and MCF7-COX2 (ER-positive, PR-positive, and HER2-negative); as well as the highly metastatic stable miRNA-overexpression cell lines MCF7-miR526b, MCF7-miR655, and SKBR3-miR526b. We used empty vector transfected cells MCF7-Mock as a control for miRNA-overexpressing cell lines. We have previously shown that there is no significant difference in miRNA expression between MCF7 and MCF7-Mock cells [13,14]. Thus, for experiments in this article, we used MCF7 as a low miRNA-expressing control cell line and MCF7-COX2 as a high miRNA-expressing cell line. We also used human breast tumor tissues to test the correlation of miRNA with *HIF-1α* expression in tumors.

### 2.1. HIF-1α Gene and Protein Expression in Normoxia

#### 2.1.1. HIF-1α Gene Expression in Various Breast Cancer Cell Lines

**Figure 1:**
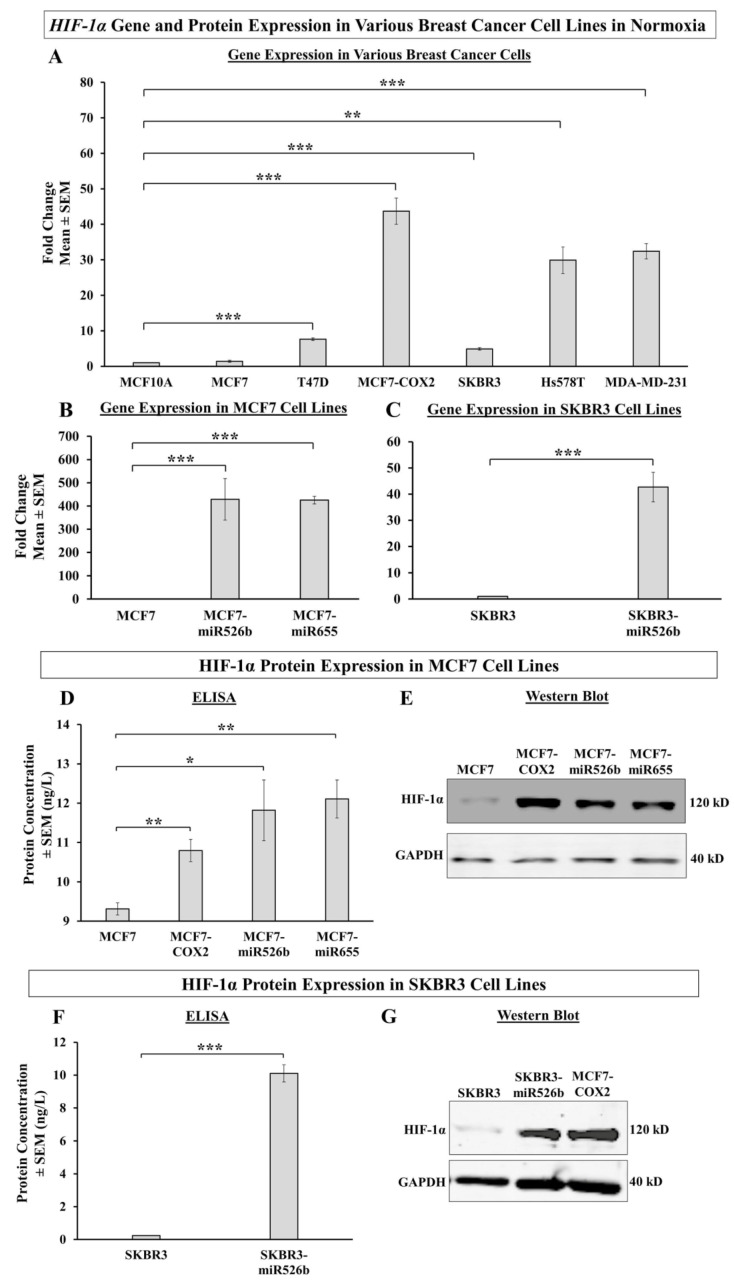
HIF-1α mRNA and protein expression in various breast cancer cell lines in normoxic conditions: (**A**) *HIF-1α* mRNA expression in various breast cancer cell lines (T47D, SKBR3, MCF7-COX2, Hs578T, and MDA-MB-231) in comparison to mammary epithelial cell line MCF10A. (**B**,**C**) *HIF-1α* mRNA expression in miRNA-high cell lines MCF7-miR526b, MCF7-miR655, and SKBR3-miR526b compared to their respective control cell lines MCF7 and SKBR3. (**D**) HIF-1α protein expression measured with ELISA in miRNA-overexpressed cell lines (MCF7-miR526b, MCF7-miR655) and miRNA-high cell line MCF7-COX2 compared to control cell line MCF7. (**E**) Western blot analysis of total endogenous HIF-1α protein expression in MCF7, MCF7-COX2, MCF7-miR526b, and MCF7-miR655 cell lines. (**F**) HIF-1α protein expression in SKBR3 and SKBR3-miR526b measured with ELISA. (**G**) Western blot analysis showing total expression of endogenous HIF-1α protein in SKBR3 and SKBR3-miR526b cell lines, MCF7-COX2 as a positive control. Full western blots are provided in Figure S3A–C. Data are presented as the mean ± SEM of triplicate replicates; * *p* < 0.05, ** *p* < 0.01, and *** *p* < 0.001.

We measured gene expression of *HIF-1α* in a variety of breast cancer cell lines in comparison to the mammary epithelial cell line MCF10A. Poorly metastatic and miRNA-low breast cancer cell lines MCF7, SKBR3, and T47D; and highly metastatic MCF7-COX2, MDA-MB-231, and Hs578T cell lines were used. miRNA expressions in these cell lines are presented in Figure S2A,B [13,14]. MDA-MB-231, MCF7-COX2, and Hs578T cell lines show very high and significant upregulation of *HIF-1α* compared to MCF10A and T47D, MCF7, and SKBR3 cell lines showed lower expressions of *HIF-1α* (Figure 1A). We observed that MCF7 and SKBR3 cell lines had the lowest levels of *HIF-1α*, and also miRNA expression. In normoxia, MCF7-miR526b and MCF7-miR655 cell lines show an extremely significant upregulation of *HIF-1α* gene expression compared to MCF7 (Figure 1B) and SKBR3-miR526b cells showed an extremely significant increase in *HIF-1α* gene expression compared to SKBR3 (Figure 1C).

#### 2.1.2. HIF-1α Protein Expression in Various Breast Cancer Cell Lines

We performed an enzyme-linked immunosorbent assay (ELISA) to test HIF-1α protein expression in the stable miRNA-overexpressed cell lines MCF7-miR526b, MCF7-miR655, and SKBR3-miR526b, as well as the naturally miRNA-high cell line MCF7-COX2, in comparison to their respective controls. HIF-1α protein levels were significantly increased in MCF7-miRNA-high cell lines compared to MCF7 cells, with MCF7-COX2 cells showing a significant but moderate increase and MCF7-miR526b and MCF7-miR655 cell lines showing high upregulation (Figure 1D). SKBR3-miR526b cells also show a significant increase in HIF-1α protein expression compared to SKBR3 cells (Figure 1F). miRNA-overexpression very significantly enhances HIF-1α expression in both ER-positive MCF7 cells and HER2-positive SKBR3 cells.

Total endogenous HIF-1α protein expression was measured with western blot analysis, data showing high expression of HIF-1α total protein in the MCF7-miR526b and MCF7-miR655 (Figure 1E) and SKBR3-miR526b (Figure 1G) cell lines compared to control miRNA low MCF7 and SKBR3 cell lines, respectively. Endogenous HIF-1α protein expression further supports results recorded with HIF-1α ELISA. 

### 2.2. Induction of Hypoxia Using CoCl_2_

To mimic the effect of a hypoxia chamber, we used CoCl_2_ to induce hypoxia as described in other publications [28,29,30,31]. We conducted a CoCl_2_ treatment dose-response assay using *HIF-1α* gene expression fold changes (Figure S1) and selected 150 μM for further experiments. It should also be noted that during CoCl_2_ treatment, we observed changes in cell density. We seeded an average of 6000 cells per well in a six-well plate and observed an increase in cell density in CoCl_2_-treated cells (Figure S4), showing that the CoCl_2_ treatment we selected was not toxic to the cells. 

#### 2.2.1. HIF-1α Gene Expression in Hypoxia

**Figure 2:**
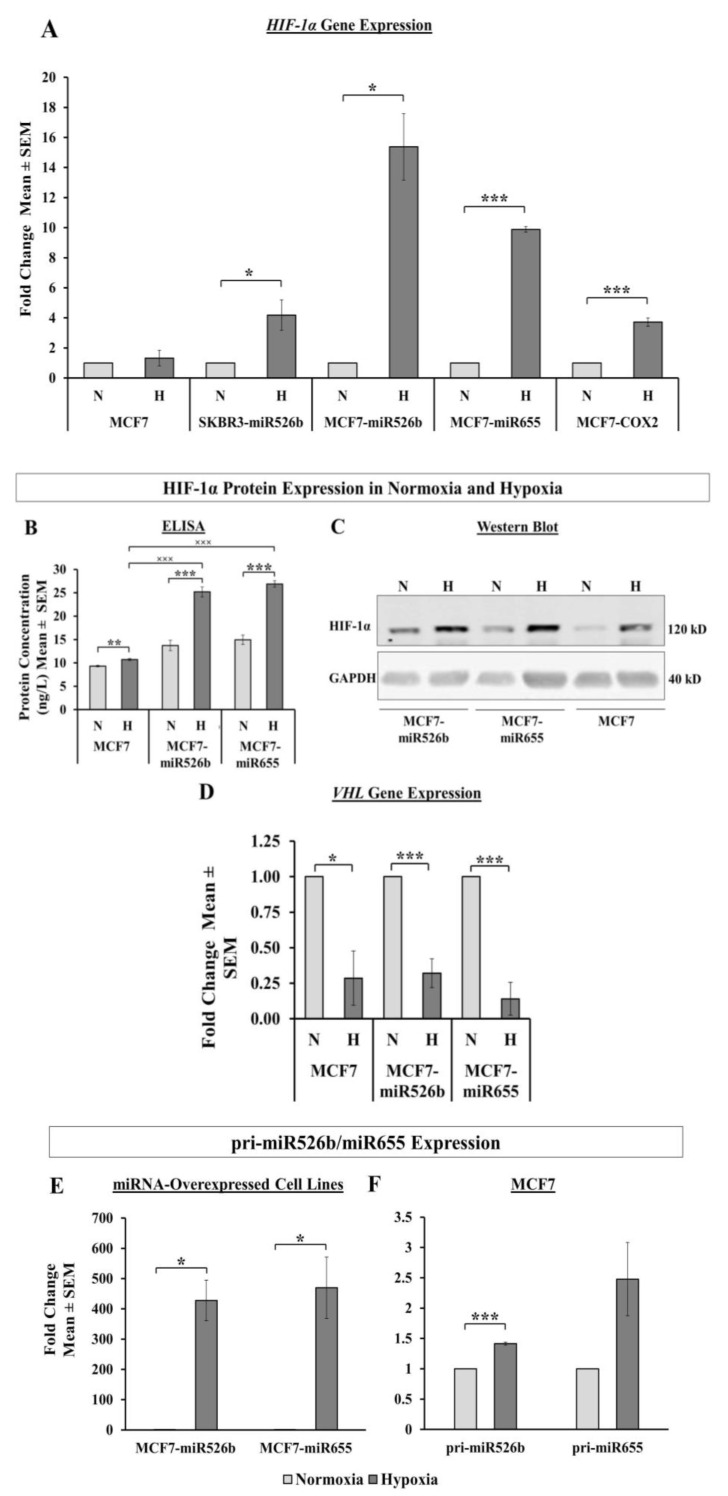
Induction of hypoxia using CoCl_2_: In all figures, ‘N’ indicates normoxia and ‘H’ indicates hypoxia. (**A**) Gene expression of *HIF-1α* in MCF7, SKBR3-miR526b, MCF7-miR526b, MCF7-miR655, and MCF7-COX2 cell lines under normoxic and hypoxic conditions measured using qRT-PCR. (**B**) Protein levels of HIF-1α in MCF7, MCF7-miR526b, and MCF7-miR655 cell lines measured using ELISA. (**C**) Total HIF-1α protein expression in both hypoxia and normoxia were measured with western blots. Complete western blots are presented in Figure S3D. (**D**) *VHL* gene expression in MCF7, MCF7-miR526b, and MCF7-miR655 cell lines measured via qRT-PCR. (**E**) Pri-miRNA expression in MCF7-miR526b and MCF7-miR655 cells in normoxia and hypoxia. (**F**) Pri-miRNA expression in MCF7 cells in normoxia and hypoxia. Data are presented as the mean ± SEM of triplicate biological replicates; * *p* < 0.05, ** *p* < 0.01, *** *p* < 0.001, and xxx indicates *p* < 0.001. * Also indicates comparison between normoxia and hypoxia of the same cell line and x indicates comparison between cell lines only in a hypoxic condition.

We used qRT-PCR to analyze *HIF-1α* gene expression in MCF7, MCF7-miR526b, MCF7-miR655, SKBR3-miR526b, and MCF7-COX2 cell lines in hypoxia, and considered sterile H_2_O treatment as the control or “normoxia.” *HIF-1α* gene expression was significantly upregulated in all cell lines except MCF7 in hypoxia compared to normoxia (Figure 2A). It should be noted, however, that miRNA-overexpressing MCF7 cell lines (MCF7-miR526b, MCF7-miR655) showed the greatest upregulation of *HIF-1α*. Thus, we decided to test hypoxia-enhanced functions in miRNA-overexpressing MCF7-miR526b and MCF7-miR655 cell lines compared to MCF7 cell lines. MCF7-COX2 showed the highest expression of *HIF-1α* expression in normoxia, thus, CoCl_2_ treatment could only moderately, but very significantly increase *HIF-1α* expression. The increase in *HIF-1α* expression in the SKBR3-miR526b cell line from normoxia to hypoxia was also modest; however, this could be the effect of HER2-positivity, which warrants further investigation.

#### 2.2.2. HIF-1α Protein Expression in Hypoxia

We performed an enzyme-linked immunosorbent assay (ELISA) to specifically test HIF-1α protein expression in MCF7, MCF7-miR526b, and MCF7-miR655 cells in hypoxia and normoxia. Microplate ELISA analysis showed that there was a significant increase in HIF-1α protein levels in all ER-positive cell lines in hypoxia compared to normoxia; however, this increase was most significant in MCF7-miR526b and MCF7-miR655 cell lines (Figure 2B). Within the hypoxic condition, we compared MCF7 and miRNA-high cell lines and both miRNA-high cell lines demonstrated a very significant increase in HIF-1α protein expression compared to MCF7 cells, indicating some form of direct involvement of miRNA in hypoxia (Figure 2B). 

We also conducted a western blot analysis of HIF-1α protein expression in MCF7, MCF7-miR526b, and MCF7-miR655 cell lines in hypoxia to measure change in total HIF-1α protein expression. For all cell lines, CoCl_2_ treatment enhanced HIF-1α expression in hypoxia compared to normoxia (Figure 2C). This enhancement of HIF-1α protein expression further confirms that CoCl_2_ treatment induces hypoxia in breast cancer.

#### 2.2.3. Analysis of VHL Gene Expression in Hypoxia

HIF-1α protein stability is dependent on *VHL*, a tumor suppressor gene that downregulates HIF-1α. We identified that *VHL* gene expression was significantly decreased in hypoxia compared to normoxia in all cell lines, with the most significant change occurring in the miRNA-high cell lines (Figure 2D). Thus, CoCl_2_ treatment successfully increased *HIF-1α* expression and downregulated *VHL* expression. 

#### 2.2.4. Hypoxia Enhances miRNA Expression

Both MCF7-miR526b and MCF7-miR655 cells showed a very significant increase in primary miRNA (pri-miRNA) expression in normoxia compared to MCF7 cells (Figure S2C,D). Since we observed an increase in HIF-1α expression in hypoxia, we wanted to determine if miRNA expression was also increased in hypoxia. Pri-miR526b expression was significantly increased in hypoxia compared to normoxia in MCF7-miR526b cells. Similarly, pri-miR655 expression was significantly increased in hypoxia in MCF7-miR655 cells compared to normoxia (Figure 2E). Most prominent changes were recorded in MCF7 cells, which showed an extremely significant increase in pri-miR526b expression and a marginal increase in pri-miR655 expression in hypoxia compared to normoxia (Figure 2F).

### 2.3. Hypoxia Induces Oxidative Stress 

Previously, we have shown that miRNA overexpression in MCF7 cells and cell-free conditioned media from miRNA-high cells (MCF7-miR526b and MCF7-miR655) induces oxidative stress [27]. In the current study, we tested if hypoxia can further stimulate oxidative stress in miRNA-high cells. Here we show data for only 150 μM CoCl_2_ treatment, since we found that the 150 μM concentration of CoCl_2_ induced maximum hypoxia (Figure S1). 

#### 2.3.1. Fluorescence Microscopy Assay to Measure Cellular Fluorescence

**Figure 3:**
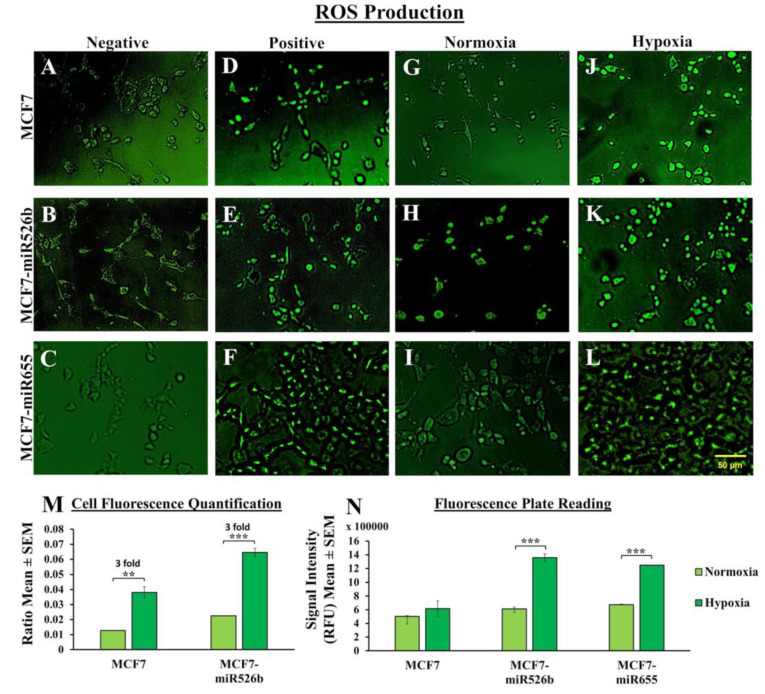

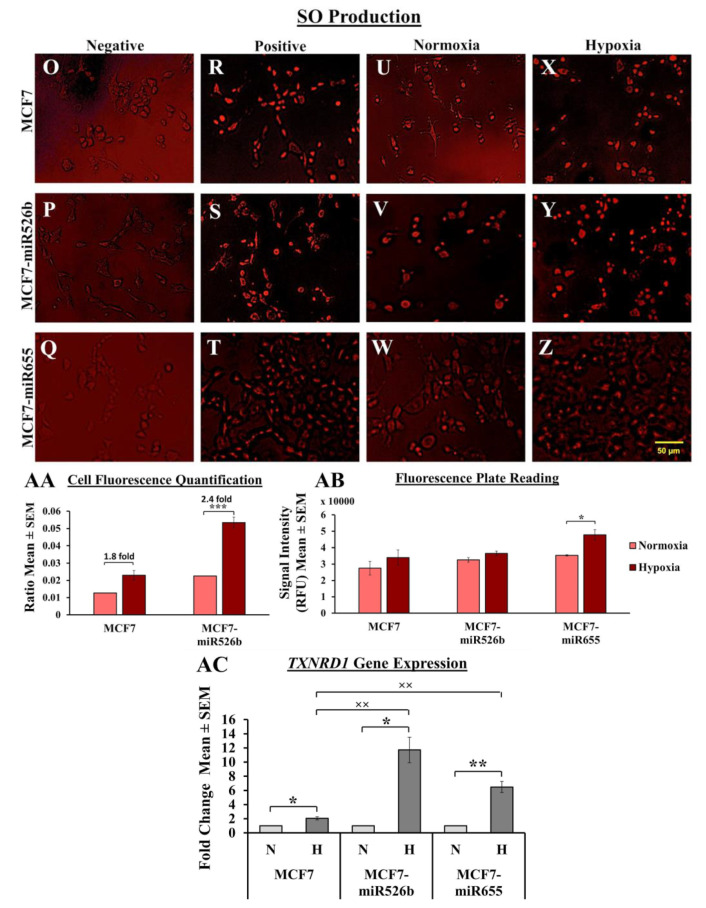
Fluorescence microscopy and fluorescence microplate assays to quantify ROS and SO production: MCF7, MCF7-miR526b, and MCF7-miR655 cells in (**A**–**C**) negative control, (**D**–**F**) positive control, (**G**–**I**) in normoxia, and (**J**–**L**) in hypoxia under the green filter for total ROS detection. (**M**) Quantification ratios of MCF7 and MCF7-miR526b cells positive for ROS in normoxia and hypoxia. (**N**) Fluorescence microplate assay to quantify total ROS production in MCF7, MCF7-miR526b, and MCF7-miR655 cells. Fluorescent SO-positive MCF7, MCF7-miR526b, and MCF7-miR655 cells (**O**–**Q**) in negative control, (**R**–**T**) in positive control, (**U**–**W**) in normoxia, and (**X**–**Z**) in hypoxia under the red filter for total SO detection. (**AA**) Quantification ratios of MCF7 and MCF7-miR526b cells positive for SO in normoxic and hypoxic conditions. (**AB**) Fluorescence microplate assay to quantify total SO production in MCF7, MCF7-miR526b, and MCF7-miR655 cells. (**AC**) Gene expression of *TXNRD1* measured with qRT-PCR. ‘N’ indicates normoxia, and ‘H’ indicates hypoxia. Scale bar represents 50 μM. (**M**,**N**,**AA**,**AB**,**AC**) Data are presented as the mean ± SEM of triplicate biological replicates; * *p* < 0.05, ** *p* < 0.01, *** *p* < 0.001, and xx indicates *p* < 0.01. * Also indicates comparison between normoxia and hypoxia of the same cell line and x, indicates comparison between cell lines only in a hypoxic condition.

Fluorescence microscopy images showing ROS (green) and SO (red) production in MCF7, MCF7-miR526b, and MCF7-miR655 cells in hypoxia and normoxia, with quantification presented in Figure 3. Negative controls of MCF7 (Figure 3A,O), MCF7-miR526b (Figure 3B,P), and MCF7-miR655 (Figure 3C,Q) as well as positive controls of MCF7 (Figure 3D,R), MCF7-miR526b (Figure 3E,S), and MCF7-miR655 (Figure 3F,T) were used to normalize fluorescence-positive cell quantifications. Only bright fluorescent cells normalized to the negative control of respective cell lines were considered for quantifications. We observed a significant increase in ROS and SO in MCF7-miR526b cells in hypoxia (Figure 3K,Y) compared to normoxia (Figure 3H,V). There was an increase in ROS and SO producing cells in MCF7 cells in hypoxia (Figure 3J,X) compared to normoxia (Figure 3G,U) as well. Quantitative data show that ROS production in hypoxia was increased three-fold in both MCF7 and MCF7-miR526b cells (Figure 3M); however, SO production in hypoxia was enhanced 1.8- and 2.4-fold in MCF7 and MCF7-miR526b cells, respectively, compared to normoxia (Figure 3AA). Images of MCF7-miR655 cells in hypoxia (Figure 3L,Z) and normoxia (Figure 3I,W) evidently show higher expression of ROS and SO in hypoxia; however, quantitative data for MCF7-miR655 are not presented.

#### 2.3.2. Fluorescence Microplate Assay to Measure Total Fluorescence

After finding cellular fluorescence in the microscopy assay, we measured total ROS and SO production using fluorescence microplate assays as described in previous studies [27]. Total ROS production was marginally but non-significantly increased in MCF7 in hypoxia compared to normoxia, whereas we recorded a significant increase in ROS production by both MCF7-miR526b and MCF7-miR655 in hypoxia (Figure 3N). Additionally, there was a marginal increase in total SO production by MCF7 cells and MCF7-miR526b cells, but only in MCF7-miR655 we observed a significant increase in SO production in hypoxia compared to normoxia (Figure 3A,B). Fluorescence microplate assays evidently show that hypoxia only enhances total ROS (Figure 3N) and SO production (Figure 3A,B) in miRNA-high cells.

#### 2.3.3. Overexpression of TXNRD1 

*TXNRD1* is a marker associated with oxidative stress. We previously showed that MCF7-miR526b and MCF7-miR655 cell lines overexpress *TXNRD1* compared to MCF7 cells in normoxia [27]. In the current study, we measured changes in *TXNRD1* expression in hypoxia in MCF7, MCF7-miR526b, and MCF7-miR655 cell lines to determine if hypoxia further enhances *TXNRD1* expression. We observed that hypoxia promotes *TXNRD1* expression in all three cell lines; however, MCF7-miR526b and MCF7-miR655 cells showed a very significant increase compared to normoxia (Figure 3AC). Additionally, we compared *TXNRD1* expression in hypoxia between cell lines and found that *TXNRD1* expression is very significantly higher in miRNA-high cells in comparison to MCF7 (Figure 3AC). Collectively, our results strongly suggest that hypoxia further enhances oxidative stress induction in miRNA-high cells.

### 2.4. Hypoxia Promotes EMT in miRNA-High Cells 

Previously, we have indicated that the overexpression of miR526b and miR655 induces EMT phenotypes in MCF7 and SKBR3 cells [13,14]. Furthermore, we have shown cell-free secretions from miRNA-high cells induce migration of vascular endothelial cells [3]. Thus, we wanted to investigate the role of hypoxia in promoting the EMT of miRNA-high cell lines. We used qRT-PCR to measure the gene expressions of mesenchymal markers (*VIM*, *TWIST1*, *SNAIL*) and the epithelial marker *CDH1*, and proceeded to perform a migration assay on miRNA-high cells in normoxic and hypoxic conditions. In hypoxia, we observed miRNA-high cell lines mimicking vascular properties, forming tube-like structures on growth factor-reduced Matrigel. 

#### 2.4.1. Hypoxic Condition Regulates EMT Markers Expression in Cancer Cells

**Figure 4:**
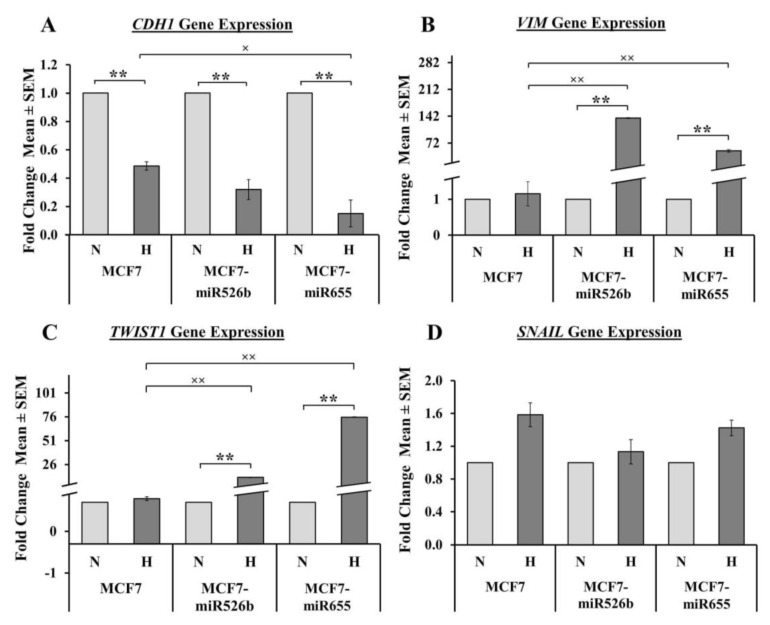
Expression of EMT markers in MCF7, MCF7-miR526b, and MCF7-miR655 cell lines: ‘N’ indicates normoxia, and ‘H’ indicates hypoxia. (**A**) Epithelial marker *CDH1* gene expression. (**B**) Gene expression of mesenchymal marker *VIM*. (**C**) Gene expression of mesenchymal marker *TWIST1*. (**D**) Gene expression of mesenchymal marker *SNAIL*. Data are presented as the mean ± SEM of quadruplicate replicates; ** *p* < 0.001, x indicates *p* < 0.05 and xx indicates *p* < 0.001. * Also indicates comparison between normoxia and hypoxia of the same cell line and x, indicates comparison between cell lines only in a hypoxic condition.

We measured mRNA expression of the epithelial marker *CDH1* and the mesenchymal markers *VIM*, *TWIST1*, and *SNAIL* in MCF7 and miRNA-high cells in both normoxic and hypoxic conditions using qRT-PCR. We observed a significant downregulation of the epithelial marker *CDH1* in all cell lines in hypoxia compared to normoxia (Figure 4A). Moreover, we observed an extremely significant upregulation of the mesenchymal markers *VIM* and *TWIST1* in MCF7-miR526b and MCF7-miR655 cells in hypoxia compared to normoxia (Figure 4B,C). For all cell lines, there was a marginal, but non-significant increase in *SNAIL* expression in hypoxia compared to normoxia (Figure 4D). Although there was a marginal increase in mesenchymal marker expression in MCF7 cells in hypoxia compared to normoxia, these changes were not significant (Figure 4B–D). We also compared *CDH1*, *VIM*, and *TWIST1* expressions in MCF7-miR526b and MCF7-miR655 cell lines compared to MCF7 only in hypoxia. We found a significant downregulation of *CDH1* in MCF7-miR655 cells in hypoxia compared to MCF7 cells in hypoxia (Figure 4A) and an extremely significant upregulation of *VIM* and *TWIST1* in miRNA-high cells (Figure 4B,C). 

#### 2.4.2. Hypoxic Condition Promotes Migration of miRNA-High Cells 

**Figure 5:**
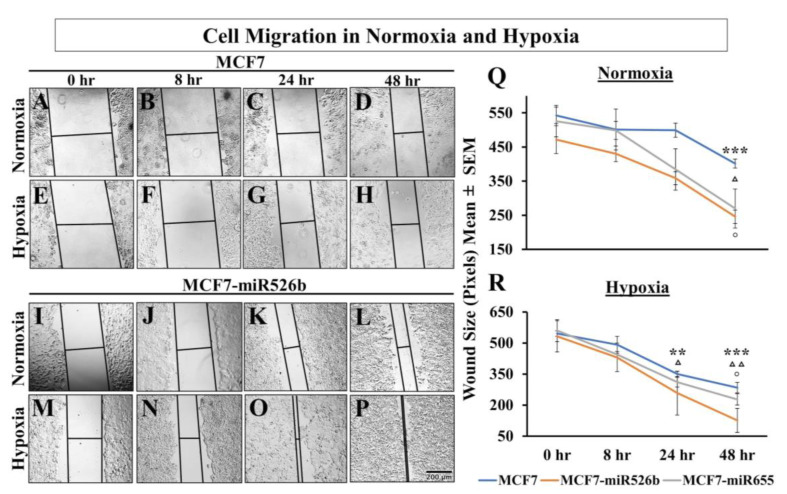
Cell migration in normoxia and hypoxia: baseline scratches represented by black lines at 0, 8, 24, and 48 h time points. Representative images of MCF7 are presented in (**A**–**D**) normoxia and (**E**–**H**) hypoxia. Representative images for MCF7-miR526b are presented in (**I**–**L**) normoxia and (**M**–**P**) hypoxia. Scale bar represents 200 µM. Wound size measured in pixels. (**Q**) Mean wound size in normoxic conditions over 0–48 h. (**R**) Mean wound size in hypoxic conditions over 0–48 h. Data are presented as the mean ± SEM of quadruplicate biological replicates; ** *p* < 0.01 and *** *p* < 0.001. * = MCF7, ◦ = MCF7-miR526b, ∆ = MCF7-miR655.

miRNA overexpression induces cell migration and invasion of both MCF7 and SKBR3 cell lines [13,14]. We previously showed that cell-free secretions from MCF7-miR526b and MCF7-miR655 cells promote migration of HUVECs [3]. Here, we tested changes in cell migration in hypoxia of MCF7, MCF7-miR526b, and MCF7-miR655 cell lines by conducting a scratch-wound cell migration assay over 48 h. In normoxia, both MCF7 (Figure 5A–D) and MCF7-miR526b (Figure 5I–L) migrated; however, MCF7-miR526b cells migrated faster through the various time points (Figure 5Q). In hypoxia, MCF7-miR526b cells (Figure 5M–P) significantly migrated and closed the wound by 24 h and completely sealed the wound by 48 h, whereas this movement was limited for MCF7 cells (Figure 5E–H). Quantitative data are presented in Figure 5R for hypoxia. MCF7-miR655 showed similar phenotypes, image data are presented in Figure S5 and quantitative data are presented in Figure 5R. Hypoxic conditions very significantly increased miRNA-induced cell migration in miRNA-high cells. 

### 2.5. Hypoxia Promotes Inflammatory Gene Expression and Vascular Mimicry in miRNA-High Cells

We have previously shown that miR655 overexpression in MCF7 cells promotes COX-2 expression (Figure S2E) [14] and proposed that this could be via *NFκB1* upregulation in the ER-positive breast cancer cell line [13,14]. We have also shown that COX-2 stimulates the production of PGE2 (prostaglandin E₂), which activates EP4 and consequently activates the PI3K/Akt pathway and promotes breast cancer angiogenesis and lymphangiogenesis [32,33,34,35]. Furthermore, we have indicated the overexpression of miR526b and miR655 downregulates *PTEN* [3], resulting in the upregulation of *VEGF*s. Here we investigated if hypoxia can regulate miRNA functions following the same signaling pathways. To establish a link between miRNA, HIF-1α, and the COX-2/EP4/PI3K/Akt pathway, we measured *NFκB1*, *COX-2*, *EP4*, and *VEGFA* gene expression in breast cancer cell lines, in both normoxia and hypoxia. We used qRT-PCR to measure gene expression of *NFκB1*, *COX-2*, and *EP4*. 

#### 2.5.1. Hypoxia Promotes Expression of NFκB1, COX-2, and EP4

**Figure 6:**
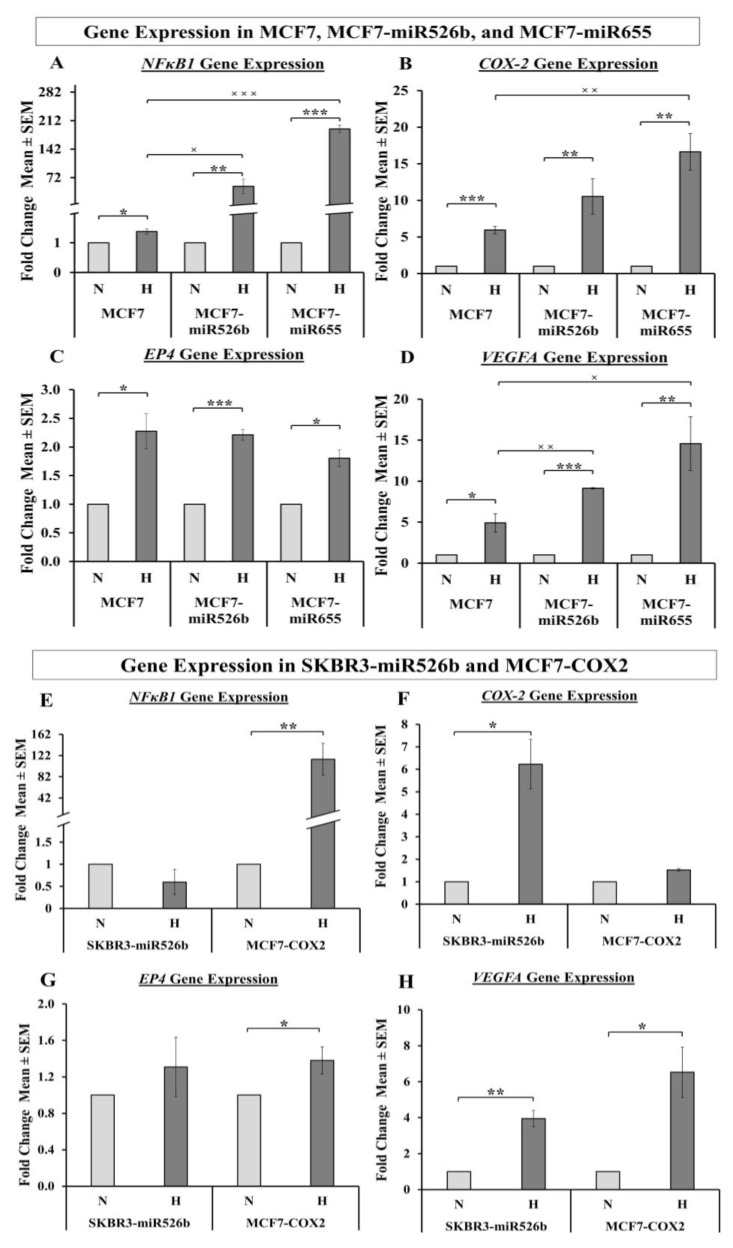
*NFκB1*, *COX-2*, *EP4*, and *VEGFA* gene expression: ‘N’ indicates normoxia, and ‘H’ indicates hypoxia. (**A**) *NFκB1*, (**B**) *COX-2*, (**C**) *EP4*, and (**D**) *VEGFA* represents gene expression in MCF7, MCF7-miR526b, and MCF7-miR655 cell lines. (**E**) *NFκB1*, (**F**) *COX-2*, (**G**) *EP4*, and (**H**) *VEGFA* gene expression in SKBR3-miR526b and MCF7-COX2 cell lines. Data are presented as the mean ± SEM of quadruplicate replicates; * *p* < 0.05, ** *p* < 0.01 and *** *p* < 0.001. x indicates *p* < 0.05, xx indicates *p* < 0.01 and xxx indicates *p* < 0.001. * Also indicates comparison between normoxia and hypoxia of the same cell line and x indicates comparison between cell lines only in hypoxia.

While *NFκB1* expression significantly increased in all three MCF7 cell lines in hypoxia compared to normoxia, this increase was more prominent and significant in MCF7-miR526b and MCF7-miR655 cell lines (Figure 6A). We also compared the gene expression of *NFκB1* in MCF7-miR526b and MCF7-miR655 cell lines in hypoxia with MCF7 cells and observed that both miRNA-high cell lines show a significant increase in *NFκB1* expression compared to MCF7 cells (Figure 6A). We tested the change in *NFκB1* expression in two other miRNA high cell lines SKBR3-miR526b and MCF7-COX2. In SKBR3-miR526b cells under hypoxia compared to normoxia there was no change in *NFκB1* expression. However, we noted a significant increase in *NFκB1* expression in the ER-positive, miRNA-high MCF7-COX2 cells in hypoxia compared to normoxia (Figure 6E). *COX-2* gene expression was significantly increased in hypoxia compared to normoxia in all cell lines except MCF7-COX2; data for MCF7, MCF7-miR526b, MCF7-miR655 are presented in Figure 6B and data for SKBR3-miR526b and MCF7-COX2 are presented in Figure 6F. It should be noted, however, that *COX-2* overexpression in hypoxia was larger in miRNA-high cell lines (Figure 6B,F) compared to that of MCF7-COX2, which only showed a marginal increase in *COX-2* expression. This could be due to the fact that MCF7-COX2 cells are already high in COX-2, so hypoxia could only marginally enhance *COX-2* expression. However, miRNA-overexpression enhances *COX-2* expression, and hypoxia further enhances this in both MCF7 and SKBR3 miRNA-overexpressed cell lines. MCF7-miR655 cells in hypoxia exhibited a very significant increase in *COX-2* expression compared to MCF7 cells in hypoxia (Figure 6B). *EP4* gene expression was significantly higher in MCF7, MCF7-miR526b, and MCF7-miR655 cell lines (Figure 6C), as well as in SKBR3-miR526b and MCF7-COX2 cell lines (Figure 6G) in hypoxia compared to normoxia. 

#### 2.5.2. Hypoxia Promotes VEGFA Gene Expression

We previously showed that miRNA overexpression in MCF7 cell lines enhanced *VEGFA* mRNA and protein production in both miRNA-overexpressed cell lines [3]. Here we analyzed mRNA expression of *VEGFA* in MCF7, MCF7-miR526b, MCF7-miR655, SKBR3-miR526b, and MCF7-COX2 cell lines in hypoxia and normoxia using qRT-PCR. *VEGFA* expression was increased in hypoxia for all cell lines, but this increase was highest in miRNA-high cell lines. Data for MCF7, MCF7-miR526b, and MCF7-miR655 are presented in Figure 6D and data for MCF7-COX2 and SKBR3-miR526b are presented in Figure 6H. In hypoxic conditions, miRNA-high cells show a significant increase in *VEGFA* expression in MCF7-miR526b and MCF7-miR655 cell lines compared to MCF7 cells (Figure 6D). Thus, hypoxia further enhanced vascular gene expression in miRNA-high cells.

#### 2.5.3. Hypoxia Promotes Vascular Mimicry

Tumor cells mimic the properties of vascular endothelial cells and form tube-like vascular structures in a process called vascular mimicry, which show an overexpression of VEGF. MCF7 cells are poorly metastatic cell lines with no vascular properties and cannot form tubes on growth factor-reduced Matrigel. We previously showed that cell-free secretions from MCF7-miR526b and MCF7-miR655 cell lines promote tube formation in HUVECs and produce VEGFs [3]; however, we have never tested the tube formation abilities of miRNA-overexpressing cells in hypoxia. Here we tested the vascular mimicry properties of MCF7, MCF7-miR526b, and MCF7-miR655 cell lines in hypoxia and normoxia. In normoxia, we found that only MCF7-miR526b and MCF7-miR655 cell lines can form tube-like structures at 24 and 48 h, but MCF7 cannot (images in Figure S6A–F, data in Figure S6G–L). In hypoxic conditions, we observed tube-like structures in MCF7 and miRNA-high cells, but miRNA-high cells produced a significantly higher number of complete tubes compared to MCF7 (images in Figure S6M,Q,U, data in Figure S6G–L). These results further confirmed that hypoxic conditions enhance vascular properties in ER-positive breast cancer cells and that hypoxia enhances vascular mimicry properties of miRNA-high cells.

### 2.6. Inhibition of Hypoxia-Enhanced Functions in miRNA-High Cells

The above results indicate that hypoxia enhances *COX-2*, *EP4*, and *NFκB1* expression. We have previously shown that miRNA expression and miRNA-induced functions can be abrogated with a COX-2 inhibitor (Celecoxib, CEL), an EP4 antagonist (ONO-AE3-208, ONO), and an irreversible PI3K/Akt inhibitor (Wortmannin, WM) [3,13,14]. Here we wanted to investigate the effect of inhibition of COX-2/EP4/PI3K/Akt signaling pathways on hypoxia-enhanced miRNA functions and miRNA expression. To investigate the direct involvement of miR526b and miR655 in hypoxia, we would also need to knockdown miR526b/miR655 in aggressive breast cancer cells in normoxia and hypoxia and test if that would inhibit miRNA induced functions. However, we were unable to conduct miRNA-knockdown experiments. 

#### 2.6.1. Pri-miRNA and HIF-1α Gene Expression Abrogated with COX-2 Inhibitor and EP4 Antagonist 

**Figure 7:**
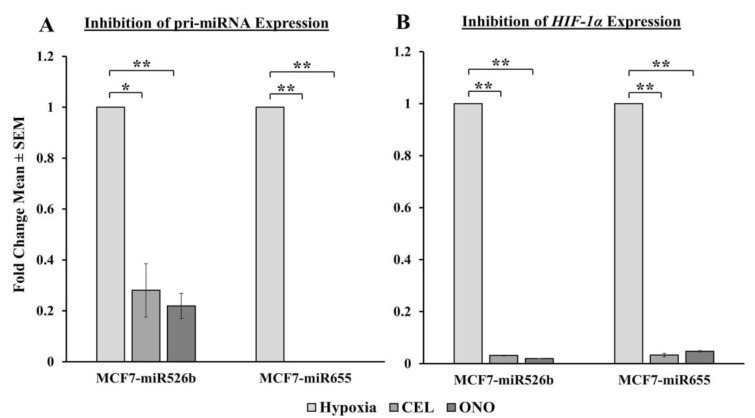
Pri-miR526b, pri-miR655, and *HIF-1α* gene expression in hypoxia and in hypoxia with inhibition: (**A**) Inhibition of pri-miR526b and pri-miR655 gene expression in hypoxia. (**B**) Inhibition of *HIF-1α* gene expression in hypoxia. Data are presented as the mean ± SEM of quadruplicate replicates; * *p* < 0.01 and ** *p* < 0.001.

We wanted to determine if pri-miR526b, pri-miR655, and *HIF-1α* gene expression could be reduced in hypoxia with CEL and ONO treatments. Expression of pri-miR526b in hypoxic MCF7-miR526b cells was significantly reduced by non-specific COX-2 inhibitor Celecoxib (CEL), and very significantly reduced by EP4 receptor-specific antagonist ONO-AE3-208 (ONO) treatments. Expression of pri-miR655 in hypoxic MCF7-miR655 cells was very significantly downregulated by both CEL and ONO compared to cells in hypoxic condition only (Figure 7A). We then measured *HIF-1α* expression in cells in hypoxia that had been treated with inhibitors. Both MCF7-miR526b and MCF7-miR655 cells in hypoxia show a very significant decrease in *HIF-1α* gene expression when treated with CEL and ONO, in comparison to hypoxic cells without inhibitor treatment (Figure 7B). These results strongly suggested that enhanced functions and increase in inflammatory gene expression in miRNA-high cell lines during hypoxia is following COX-2/EP4 signaling [3,13,14]. To test the involvement of PI3K/Akt cell signaling in hypoxia-induced functions, we use an irreversible inhibitor WM to block miRNA functions, which we previously showed to strongly regulate miRNA functions in breast cancer [3]. 

#### 2.6.2. Inhibition of ROS/SO Production

**Figure 8:**
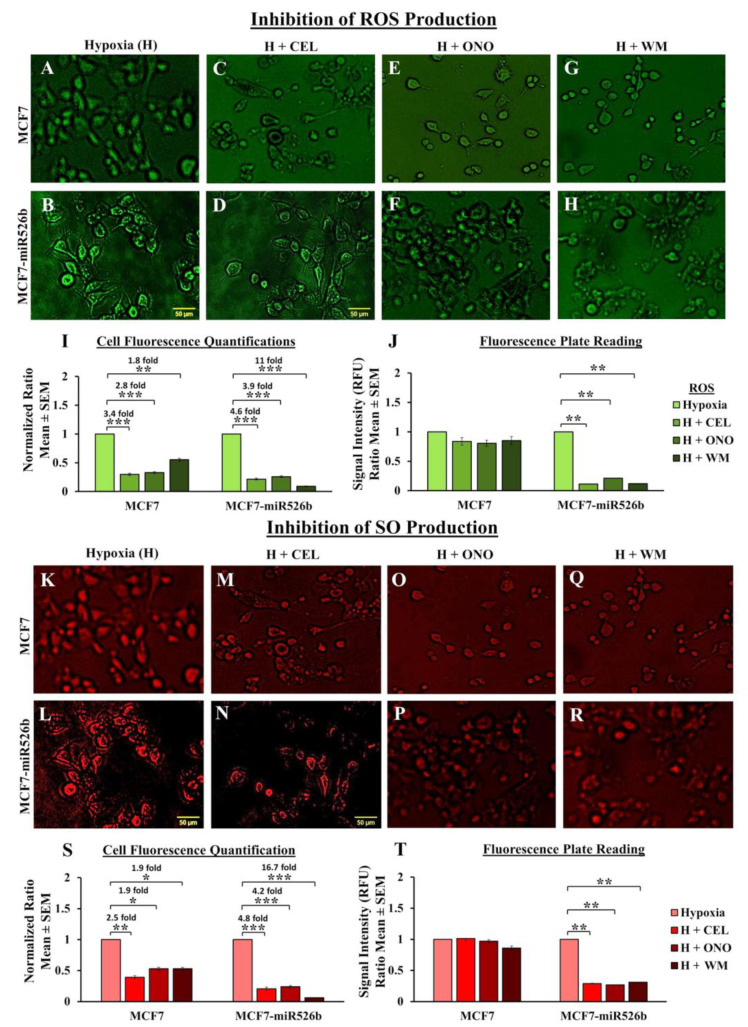
Inhibition of ROS and SO production: Representative MCF7 and MCF7-miR526b cells in (**A**,**B**) hypoxia, (**C**,**D**) hypoxia with CEL, (**E**,**F**) hypoxia with ONO, and (**G**,**H**) hypoxia with WM under the green filter for total ROS detection. (**I**) Quantification ratios for MCF7 and MCF7-miR526b cells positive for ROS. (**J**) Fluorescence microplate assay to quantify total ROS production. Fluorescent MCF7 and MCF7-miR526b cells in (**K**,**L**) hypoxia, (**M**, **N**) hypoxia with CEL, (**O**,**P**) hypoxia with ONO, and (**Q**,**R**) hypoxia with WM under the red filter for total SO detection. (**S**) Quantification ratios of MCF7 and MCF7-miR526b cells positive for SO. (**T**) Total SO production measured with fluorescence microplate assay. For all pictures, the scale bar represents 50 μM. Quantitative data are presented as the mean of three biological replicates ± SEM. * *p* < 0.05, ** *p* < 0.01 and *** *p* < 0.001.

To examine hypoxia-enhanced ROS and SO production in MCF7, MCF7-miR526b, and MCF7-miR655 cell lines. We tested if CEL, ONO, and WM could significantly block hypoxia-enhanced ROS production in all cell lines. Fluorescence images for MCF7 and MCF7-miR526b ROS-positive cells treated with various inhibitors are presented in Figure 8A,C,E,G and Figure 8B,D,F,H, respectively. Quantification for ROS-positive cell fluorescence is presented in Figure 8I. The fold difference for ROS production before and after inhibitor treatments was very prominent for MCF7-miR526b (between 3.9–11-fold) compared to MCF7 (between 1.8–3.4-fold) (Figure 8I). Both MCF7 and MCF7-miR526b cells show significantly reduced ROS-positive cells with inhibitor treatments. However, MCF7-miR526b cells in hypoxia show a sharper decrease in ROS production after inhibitor treatments than MCF7 cells, as denoted by the fold differences. We observed a very similar phenomenon with SO production. SO production in MCF7 and MCF7-miR526b cell lines in hypoxia were inhibited by CEL, ONO, and WM. Fluorescence images for MCF7 and MCF7-miR526b SO-positive cells treated with various inhibitors are presented in Figure 8K,M,O,Q and Figure 8L,N,P,R, respectively. Quantification for SO cell fluorescence is presented in Figure 8S. 

Similarly, these inhibitors blocked hypoxia-enhanced SO production in MCF7-miR526b cells (4.2–16.7-fold) compared to MCF7 (1.9–2.5-fold) (Figure 8S). We also measured inhibition of total fluorescence emission by cells treated with inhibitors. There was a marginal decrease in total fluorescence by MCF7 cells with treatments, whereas for MCF7-miR526b inhibitors could significantly abrogate hypoxia induced ROS and SO production. Total fluorescence emission measurement for ROS is presented in Figure 8J and for SO is presented in Figure 8T. Fluorescence microplate assays strongly imply that hypoxia enhances miRNAs’ promotion of ROS and SO production in miRNA-high cells. This stimulation was significantly inhibited by COX-2, EP4, and PI3K/Akt inhibitors indicate a miRNA-specific function.

#### 2.6.3. Inhibition of Cell Migration 

**Figure 9:**
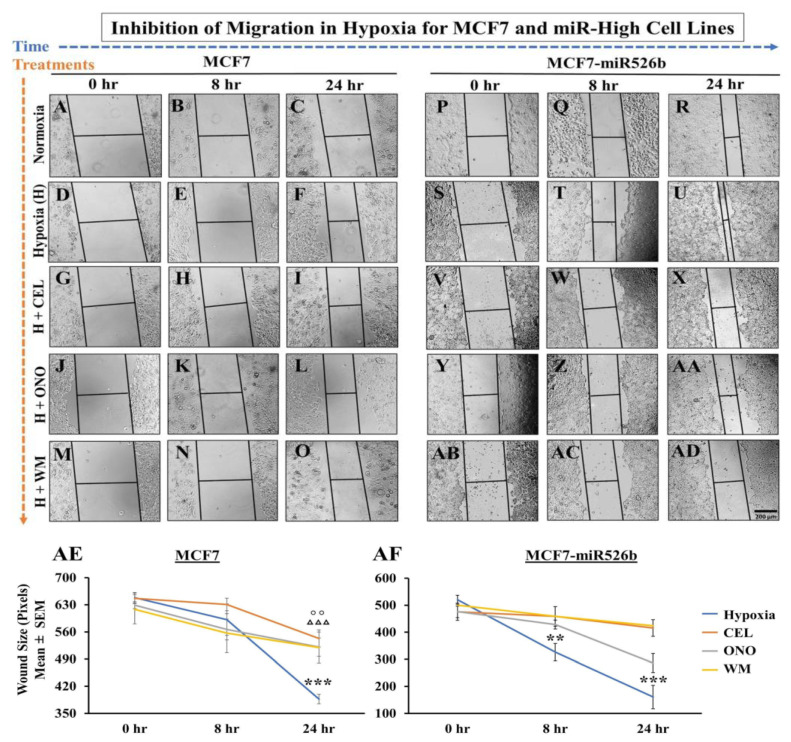
Inhibition of cell migration: Migration of cells recorded at 0, 8 and 24 h. (**A**–**C**) Representative images of MCF7 cells in normoxia, (**D**–**F**) in hypoxia, (**G**–**I**) hypoxia with CEL, (**J**–**L**) hypoxia with ONO and (**M**–**O**) hypoxia with WM. (**P**–**R**) Representative images for MCF7-miR526b in normoxia, (**S**–**U**) in hypoxia, (**V**–**X**) hypoxia with CEL, (**Y**–**AA**) hypoxia with ONO, and (**AB**–**AD**) hypoxia with WM. (**AE**) Quantitative data for the inhibition of MCF7 cell migration with inhibitors. (**AF**) Quantitative data of inhibition of hypoxia-enhanced migration of MCF7-miR526b. Data are presented as the mean ± SEM of quadruplicate biological replicates; ** *p* < 0.01 and *** *p* < 0.001. * = Hypoxia, ◦ = Celecoxib, ▵ = ONO-AE3-208.

Hypoxia promotes migration of MCF7, MCF7-miR526b, and MCF7-miR655 cell lines. In hypoxia, MCF7 cells migrated marginally (images in Figure 9D–F, quantification in Figure 9AE) compared to the control MCF7 normoxia cells (Figure 9A–C). However, miRNA-high cells migrated significantly and sealed the wound (images in Figure 9S–U, quantification in 9AF) compared to the standard MCF7-miR526b normoxia cells (Figure 9P–R). In MCF7 cells, CEL (Figure 9G–I), ONO (Figure 9J–L), and WM (Figure 9M–O) marginally reduced the wound sizes at 24 h. Quantitative data for MCF7 are presented in Figure 9AE. Hypoxia enhanced cell migration of MCF7-miR526b cells at both 8 h, and 24 h. This migration was inhibited in the presence of CEL (Figure 9V–X); ONO (Figure 9Y–AA); or WM (Figure 9AB–AD). Quantitative data are presented in Figure 9AF. Similarly, it was found that MCF7-miR655 cells in hypoxia had significantly smaller wound sizes at both 8 and 24 h (Figure S7A–C), while hypoxic cells treated with CEL (Figure S7D–F), ONO (Figure S7G–I), or WM (Figure S7J–L) showed marginally smaller wound sizes at 8 h and significantly smaller wound sizes at 24 h. Quantification for MCF7-miR655 is presented in Figure S7M. In both miRNA-high cells, while COX-2 inhibitor and EP4 antagonist could partially block cell migration; irreversible PI3K/Akt inhibitor completely blocked cell migration. These results indicate that hypoxia enhances migration of very significantly in miRNA-high cells via COX-2/EP4/PI3K/Akt pathways, which was evidently absent in miRNA low MCF7 cells. Thus, enhancement of cell migration in hypoxia is due to miRNA.

#### 2.6.4. Inhibition of Vascular Mimicry

We observed inhibition of hypoxia-enhanced tube formation by MCF7-miR526b and MCF7-miR655 cells in the presence of CEL, ONO, and WM. Images are presented in Figure S6N–P,R–T,V–X . Data are presented in Figure S6G–L. This indicates that hypoxia promotes miRNA-induced vascular mimicry phenotypes in miRNA-high cells following the same COX-2 and miRNA-induced angiogenesis and lymphangiogenesis pathways [3,33,34].

### 2.7. Linking COX-2, EP4, and PI3K/Akt Pathways with Hypoxia and miRNAs

**Figure 10:**
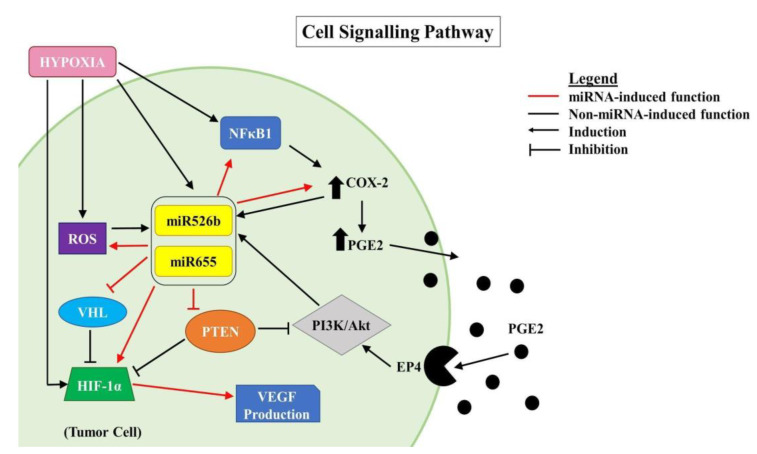
Linking COX-2, EP4, and PI3K/Akt pathways with hypoxia, miR526b, and miR655: red lines indicate functions induced by miR526b and miR655, while black lines indicate functions that are not directly induced by miRNAs. Arrows indicate induction, while T-shaped lines indicate inhibition.

We have shown that miR655 overexpression promotes *COX-2* expression in the ER-positive breast cancer cell line MCF7 (Figure S2E) [14] and here we showed that in hypoxia *COX-2* mRNA expression is enhanced (Figure 6B). We have also shown that *NFκB1* is upregulated in miRNA-high cell lines, and is significantly increased under hypoxia (Figure 6A). COX-2 stimulates the production of PGE2, which activates PGE2 receptor EP4 and consequently activates the PI3K/Akt pathway [32,33,34,35]. Moreover, we have shown that the overexpression of miR526b and miR655 upregulates VEGF expression and downregulates *PTEN* [3], a negative regulator of PI3K/Akt and HIF-1*α*. The absence of *PTEN* results in the upregulation of *HIF-1α* and *VEGFA*. In this study, we found that *VHL*, a tumor suppressor gene and negative regulator of HIF-1α, is downregulated in miRNA-high cells in hypoxic conditions, which leads to upregulation of *HIF-1α*. Hypoxia-enhanced functions could be abrogated in the presence of a COX-2 inhibitor, EP4 antagonist, or PI3K/Akt inhibitor. All of these proposed pathways are illustrated in Figure 10.

### 2.8. Bioinformatics Analysis and Regulation of PTEN and NFκB1 by miRNA

#### 2.8.1. PTEN Regulation

**Figure 11:**
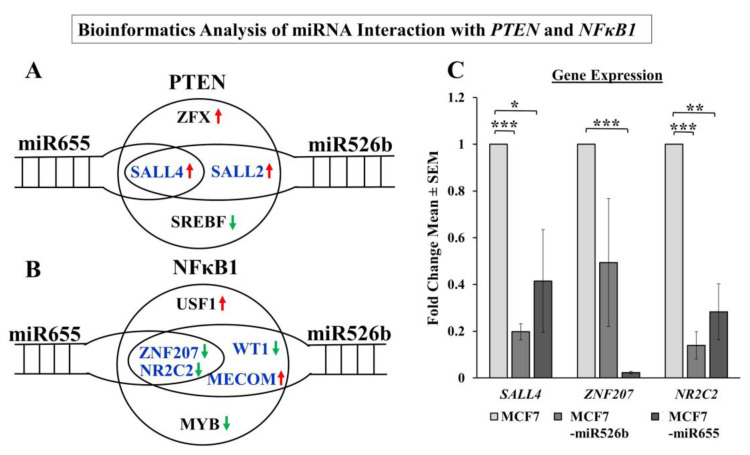
Bioinformatics analysis of the regulation of *PTEN* and *NFκB1* by miRNA-high cells: (**A**) regulation of *PTEN* by miR655 and miR526b through transcription factors *SALL2* and *SALL4*. (**B**) Regulation of *NFκB1* by miR655 and miR526b through transcription factors *MECOM*, *NR2C2*, *WT1*, and *ZNF207*. Red arrows indicate that the transcription factor upregulates gene expression, green arrows indicate that the transcription factor inhibits gene expression. (**C**) Gene expression of transcription factors targeted by both miRNAs (*SALL4*, *ZNF207*, and *NR2C2*) in MCF7, MCF7-miR526b, and MCF7-miR655 cell lines. Data are presented as the mean ± SEM of quadruplicate biological replicates. * *p* < 0.05, ** *p* < 0.001 and *** *p* < 0.0001.

We previously showed that both miR526b and miR655 regulate *PTEN* [3], and that *PTEN* downregulates HIF-1α. Although *PTEN* is not a direct target of miR526b or miR655, both miRNA modulate transcription factors that regulate *PTEN* expression. Using Targetscan via miRBase, we identified that the total number of target genes for miR526b and miR655 was 4133 and 3264, respectively [36]. From the Enrichr database, we found a total of 31 transcription factors (TFs) that regulate *PTEN* gene expression, four of which target human *PTEN*. Transcription factors *ZFX*, *SALL2*, and *SALL4* positively upregulate *PTEN*, while *SREBF* downregulates *PTEN*. Bioinformatics analysis further shows that *SALL2* is a target of miR526b and *SALL4* is directly targeted by miR655 and partially targeted by miR526b (Figure 11A), thus, we decided to measure *SALL4* expression and found an anti-correlation effect with miRNA expression. Here we observed that in both MCF7-miR526b and MCF7-miR655 cell lines, *SALL4* is significantly downregulated compared to MCF7 cells (Figure 11C), indicating an anti-correlation effect between miRNA and *SALL4*. We suggest this to be a plausible explanation for why *PTEN* is significantly downregulated with miRNA upregulation in MCF7 cells.

#### 2.8.2. NFκB1 Regulation 

Here we show that in hypoxia, miRNA-high cell lines have significant upregulation of *NFκB1* gene expression. We identified a total of 39 transcription factors (TFs) that are associated with the *NFκB1* gene, six of which were identified by the Enrichr database as TFs regulating human *NFκB1*. Amongst these six TFs, two transcription factors, *ZNF207* and *NR2C2*, negatively regulate *NFκB1*. Bioinformatics analysis shows that both *ZNF207* and *NR2C2* are the common target of both miR526b and miR655 (Figure 11B). These two TFs are significantly downregulated in miRNA-high cells compared to miRNA-low MCF7 cells (Figure 11C), indicating an anti-correlation effect between miRNAs and these TFs. The absence of these negative regulators explains why *NFκB1* expression is upregulated in miRNA-high cells. Luciferase reporter assays are needed in the future to validate that *SALL4*, *ZNF207*, and *NR2C2* are targets of miR526b and miR655. 

### 2.9. miR526b and miR655 Expression Significantly Correlates with HIF-1α Expression in Human Breast Tumors

#### 2.9.1. Ontario Tumor Bank Sample Demography

To further validate the relationship between miR526b, miR655, and *HIF-1α* expression, we tested our hypothesis on human breast cancer tissues. We collected 96 tumor tissue and 20 non-cancerous control tissue samples from the Ontario Tumor Bank and extracted total RNA, synthesized cDNA and measured gene and miRNA expressions using Taqman gene and miRNA expression assays. Demographic data of the samples are shown in Table 1. In the tumor sample set, 96.88% are female samples, 25% were considered tobacco smokers, 29.17% were considered social or occasional alcohol consumers, and 3.13% were categorized as regular drinkers. In the data set, 38.85% of tumor samples are ER-positive, and 63.54% are HER2- negative. PR-positive and PR-negative status are almost similar at 32.29% and 31.25%, respectively, and 10.42% are triple-negative breast cancer samples. In this data set, we have seven stage I tumor samples (7.29%), 45 stage II samples (46.87%), 39 stage III samples (40.63%), and five stage IV tumor samples (5.21). Control tissues are histopathologically normal with all females and 5% and 25% had smoking and alcohol habits, respectively. 

#### 2.9.2. HIF-1α and miRNA Expression in Breast Tumor and Control Tissues

**Figure 12:**
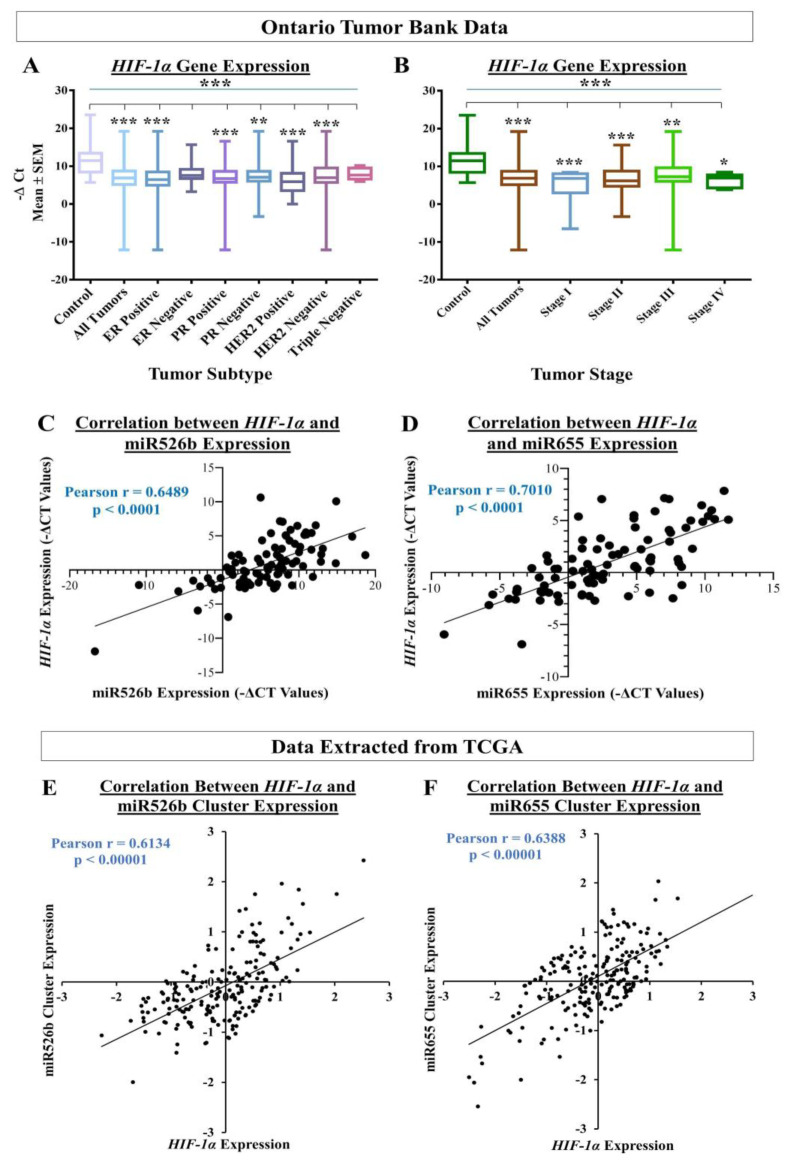
Human tumor and control tissue data: (**A**) Delta CT (∆CT) of *HIF-1α* expression in control tumor tissues, all tumor tissues, ER-positive/negative, PR-positive/negative, HER2-positive/negative, and triple-negative tumor samples. (**B**) *HIF-1α* expression in stage I, II, III, and IV tumors. (**C**) Correlation between *HIF-1α* and miR526b expression in tumor samples. (**D**) Correlation between *HIF-1α* and miR655 expression in tumor samples. In figures A and B, the *Y*-axis represents -∆CT, as smaller ∆CT values indicate higher expression. (**E**) Correlation between *HIF-1α* and miR526b cluster expression in tumor samples. (**F**) Correlation between *HIF-1α* and miR655 cluster expression in tumor samples. * *p* < 0.05, ** *p* < 0.01 and *** *p* < 0.001.

Here, we report that tumor samples showed significant upregulation of *HIF-1α* expression compared to the control tissues (Figure 12A). We also estimated that in stratified samples, *HIF-1α* expression was very significantly high in ER-positive, PR-positive, and HER2-negative breast tumors compared to the control tissues (Figure 12A). We did not find an association of *HIF-1α* expression with triple-negative breast cancer, which could be due to the fact that we had only a few triple-negative breast tumor tissues. In addition, *HIF-1α* expression was very significantly increased in stage I and II tumors, significantly increased in stage III, but only marginally high in stage IV tumors compared to the control tissues (Figure 12B). However, we have only a few stage I and stage IV tumor samples, thus, the observed association of *HIF-1α* expression is specific to stage II and stage III and the observed findings need to be validated with a larger sample set. 

We established that in the same tumor sample set, the expressions of both miRNAs are significantly high in tumor compared to control tissues, and both miRNA expressions are associated with poor patient survival [13,14]. In our previous studies, we have shown miR526b and miR655 expression to be proportionally higher in the ER-positive, PR-positive, and HER2-negative samples [3,13,14]. Here in this study, we wanted to investigate a possible link between miRNA expressions with hypoxia in breast cancer. To find any correlation between miRNA expression and *HIF-1α* expression in tumor tissues, we conducted a Pearson correlation coefficient analysis. We observed a very significant positive correlation between miR526b expression and *HIF-1α* expression (Figure 12C), and between miR655 expression and *HIF-1α* expression (Figure 12D). For miR526b and *HIF-1α*, the R-value is 0.6489, and for miR655 and *HIF-1α*, the R-value is 0.7010, showing a strong positive correlation. Due to few samples in stratified tumor subtype categories, we did not perform a correlation analysis between miRNA and *HIF-1α* in each tumor subtype and stage. This should be investigated in future studies. 

#### 2.9.3. Data Extracted from the cBioPortal Database Via TCGA 

To further validate the link between miRNA expressions with hypoxia in breast cancer, we used human breast tumor gene and miRNA expression data available in the TCGA database. We used the cBioPortal database within TCGA to extract breast cancer specific gene and miRNA expression data [37,38]. In total, we used compiled breast cancer tumor tissue data from 16 breast cancer studies included in cBioPortal. Here, we compared the *HIF-1α* mRNA expression to the mean miRNA cluster expression of either miR526b or miR655. While conducting correlation of *HIF-1α* and miRNA clusters, we excluded samples that did not have data for either miRNA or *HIF-1α* expression. As a result, we had 200 samples for the miR526b cluster analysis and 202 samples for miR655 cluster analysis, which had data for both miRNA clusters and *HIF-1α*. miR526b’s miRNA cluster contains 20 miRNAs, of which only two miRNAs, miR516a-1, and miR516a-2, had available expression data. We took the mean expression data of both of these miRNAs and presented this as miR526b cluster expression. The miR655 miRNA cluster also contains 20 miRNAs, nine of which (miR154, miR369, miR381, miR382, miR409, miR410, miR487b, miR539, and miR889) had available expression data. We took the mean of all nine miRNAs expression data and presented this as miR655 miRNA cluster expression. 

With Pearson correlation coefficient analysis, miR526b cluster expression showed a very significant correlation with *HIF-1α* expression, with an R-value of 0.6134 and *p* < 0.00001 (Figure 12E). Similarly, the average expression of miR655 cluster was also very significantly correlated with *HIF-1α* expression, with an R-value of 0.6388 and *p* < 0.00001 (Figure 12F). These data, compiled from 16 different studies, shows strong implications of miR526b/miR655 expression correlated to *HIF-1α* expression in breast cancer. These results further strengthen the notion that both miRNAs collaborate in hypoxia to promote aggressive breast cancer.

## 3. Discussion

The tumor microenvironment plays a major role in tumor growth and metastasis. An aggressively growing tumor goes through a phase of hypoxia, in which the center of the tumor mass is deprived of oxygen. In order to survive, tumor cells release growth factors and chemokines, which in turn promote angiogenesis, thus allowing the tumor to bypass apoptosis [19,30]. Hypoxia promotes angiogenesis, EMT, and oxidative stress in the tumor microenvironment [30]. Our previous studies have demonstrated the roles of miR526b and miR655 as oncogenic miRNAs, promoting aggressive breast cancer phenotypes such as cell migration, invasion, tumor associated angiogenesis, cancer stem cell induction, oxidative stress, tumor growth, and metastasis [3,13,14,27]. Involvement of miRNAs to change and modulate the tumor microenvironment to promote breast cancer metastasis is a growing field of research. Thus, in this article, we tested the interaction and change of functions in two oncogenic miRNAs, miR526b, and miR655, in hypoxia.

Both hypoxia and miRNAs have been associated with the promotion of cancer in various studies, and one has been shown to regulate the other. For instance, Bandara et al. have shown that hypoxia-enhanced miRNAs play an important role in the hypoxic adaptation of cancer cells, and have demonstrated that hypoxia is also a regulator of miRNA biogenesis [39]. Here we observed that hypoxia enhances miR526b and miR655 expression in ER-positive breast cancer cells. Another study by Bhandari et al. also shows hypoxia-enhanced miRNA dysregulation in various cancers, and identified miR133a-3p as a hypoxia-modulated miRNA [40]. Hypoxia-induced miR590-5p was shown to stimulate matrix metalloprotease activity and stimulate cell migration and invasion [41]. Conversely, Krutilina et al. discovered that miR-18a directly targets *HIF-1α*, and downregulates hypoxic gene expression [12] and in colon cancer miR22 was shown to inhibit hypoxia [42]. 

HIF-1α is a transcription factor that acts as a marker for hypoxia in cells. In this study, we observed that aggressive miR526b/miR655-overexpressing cell lines (MCF7-COX2, MCF7-miR526b, MCF7-miR655, SKBR3-miR526b) produce high HIF-1α in normoxia, while poorly metastatic miRNA-low cell lines show a significantly lesser amount of HIF-1α. These results show that even under normoxic conditions, miRNA-high cell lines are naturally high in hypoxia marker expression, indicating that these miRNAs may be involved in hypoxia in breast cancer. This is supported by Kulshreshtha et al., showing miRNA directly regulates *HIF-1α* gene expression in various cancers [43]. We also observed that in hypoxia, there is a very significant increase in HIF-1α mRNA and protein expression in miRNA-high cell lines, in particular in ER-positive cells (MCF7-miR526b, MCF7-miR655) compared to miR-low MCF7 cells, indicating that this could be an ER-specific phenomena. Additionally, CoCl_2_ treatment enhanced miR526b and miR655 expression in MCF7 cells as well, thus, an increase in HIF-1α expression in MCF7 cells could be due to miRNA expression upregulation. Furthermore, the expression of HIF-1α is partly controlled by a tumor suppressor pVHL, which tags HIF-1α and sends it for degradation under normoxic conditions [44]. We found that the VHL gene was significantly downregulated in hypoxic conditions in miRNA-high cells, hence, HIF-1α expression enhanced. This established a strong link between miRNA and hypoxia. 

Hypoxic conditions are the master regulators of oxidative stress, causing ROS production, DNA damage, promoting inflammation [45,46], and oxidative stress induces inflammatory miRNA production [39,47]. In our previous study, we have shown that miR526b and miR655 directly upregulate oxidative stress in breast cancer [27]. Here, we observed that in MCF7-miR526b and MCF7-miR655 cells, ROS and SO production is further stimulated by hypoxia. The increase in ROS and SO production is greater in the miRNA-high cell lines than the increase in MCF7 cells in hypoxia. TXNRD1 is an enzyme that regulates the production of ROS and SO and overexpression of this enzyme is an indicative marker for oxidative stress. We found that hypoxia enhanced *TXNRD1* expression in all breast cancer cell lines, but the most significant increase was found in MCF7-miR655 cells. The marginal increase in oxidative stress in MCF7 cells after CoCl_2_ treatment could be a combined effect of hypoxia and miRNA-overexpression in this cell due to hypoxia. This suggests hypoxia and miR526b and miR655 collaborate to enhance oxidative stress in breast cancer.

Hypoxia can completely reprogram tumor cells to induce EMT, and stimulate vasculogenesis to enhance cell migration [48]. We have shown that miR526b and miR655 overexpression in breast cancer cells promotes EMT, cell migration, as well as *VEGFA* upregulation [3,13,14]. Here, we identified that MCF7-miR526b and MCF7-miR655 had higher levels of the mesenchymal markers (*VIM*, *TWIST1*, *SNAIL*) and lower levels of the epithelial marker *CDH1* expression in hypoxia compared to normoxia. Mesenchymal cells are highly migratory, thus, a scratch-wound migration assay was performed and found that the scratch wound closes faster in miRNA-high cells in hypoxia compared to MCF7 cells in hypoxia. We also observed that miRNA-high cell lines show vascular mimicry and promote tube formation in hypoxia. All these phenotypes support that hypoxia enhances functions of miR526b and miR655 to promote breast cancer cell aggressiveness. 

In the past research, we have identified that in ER-positive MCF7 breast cancer cells, *COX-2* overexpression significantly upregulates the expression of miR526b and miR655. miR526b and miR655 are known to upregulate *COX-2* and *EP4* expression, and we proposed that miRNA could regulate *COX-2*/*EP4* expression through the NFκB pathway [13,14]. COX-2 activity produces PGE2, which in turn binds to the EP4 receptor. EP4 activation induces PI3K/Akt signaling, which regulates angiogenesis during embryogenesis and in breast cancer metastasis [33,35,49,50]. We have also shown that COX-2, EP4, and PI3K/Akt inhibition could abrogate miRNA-induced angiogenesis in vitro [3]. A link between miRNA regulating HIF-1α expression via PI3K/Akt signaling was shown in other tumor models as well [51]. In the current study, we show that hypoxic conditions enhance *COX-2*/*EP4* and *NFκB1* expression in ER-positive breast cancer cells, and both COX-2 inhibitor Celecoxib (CEL) and EP4 antagonist ONO-AE3-208 (ONO) significantly abrogate miRNA expression. Therefore, we attempted to block the cancer-promoting phenotypes enhanced by hypoxic conditions in miRNA-high cells. Our findings show that MCF7-miR526b and MCF7-miR655 cell migration, oxidative stress, and vascular mimicry was inhibited by the application of a COX-2 inhibitor, EP4 antagonist, and an irreversible PI3K/Akt inhibitor Wortmannin (WM). The hypoxia-enhanced functions of miRNA-high cells were inhibited to a greater extent than that of miRNA-low MCF7 cells. These results strongly suggest that, in hypoxia, COX-2/EP/PI3k/Akt signaling pathways regulate miRNA functions. However, this does not show the effect of miRNA knockdown or inhibition of miRNA expression in aggressive cell lines. While we have shown in previous studies that the knockdown of miR526b and miR655 in aggressive breast cancer cell lines reduces aggressive breast cancer phenotypes [13,14], here we were unable to test the direct effects of miRNA knockdown in hypoxia. In the future, it would be interesting to investigate the effects of miR526b and miR655 knockdown on hypoxia in breast cancer. 

We previously validated that both miR526b and miR655 target *CPEB2*, which is a tumor suppressor gene and strongly correlated with p53 expression in breast cancer [15]. We have previously shown that *PTEN* expression is downregulated in miRNA-overexpressed MCF7 cell lines [3]. *PTEN* is also a tumor suppressor that downregulates the expression of *HIF-1α* and regulates the PI3K/Akt pathway. In the absence of *PTEN*, *HIF-1α* is able to act as a transcription factor for VEGFA, increasing angiogenesis, as well as activating other pathways that promote aggressive cancer phenotypes [9]. *NFκB1* is a transcription factor frequently activated in tumors that is involved in growth, progression, and resistance to chemotherapy. Various alarmin receptors are activated by *HIF-1α*, which in turn strongly activates NFκB and pro-inflammatory pathways, furthering the progression of the malignant phenotype [52]. Here we showed that in the hypoxic conditions, *NFκB1* is upregulated most significantly in miRNA-high MCF7 cells, suggesting that miRNA induces *NFκB1* expression in hypoxic conditions. To establish miRNA-signaling pathways, we examined miRNA target genes list. 

Additionally, a bioinformatics approach was taken to determine the direct connection between miR526b and miR655 with *NFκB1* and *PTEN*. A number of transcription factors regulating *NFκB1* and *PTEN* were identified as direct or indirect targets of miRNA. *SALL2* and *SALL4* are positive regulators of *PTEN* and can regulate tumor metastasis [53,54]. In our analysis, *SALL4* expression was significantly downregulated in miRNA-high cell lines compared to MCF7. We identified *ZNF207* and *NR2C2* as transcription factors that are negative regulators of *NFκB1* are significantly downregulated in miRNA-high cells. It was shown that the ZNF207-HER2 fusion protein is oncogenic in gastric cancer [55], and NR2C2 was shown to prevent MCF7 cell proliferation in an ER dependent manner [56]. In our study in miRNA-high cells, both *ZNF207* and *NR2C2* are downregulated, and thus, *NFκB1* is upregulated. Although we were unable to conduct a true miRNA target validation using a luciferase reporter assay, our overall findings finally establish the link between miRNAs, NFκB1, COX-2, EP4, PI3K/Akt, PTEN, and HIF-1α signaling pathways. 

To assess the translational impact of the discoveries, we tested the relation between miRNA and *HIF-1α* expression using human breast cancer tissue and non-cancerous control tissues. We found that there is a significant increase in *HIF-1α* gene expression in tumor tissues compared to the control tissues. In particular, ER-positive, PR-positive, and HER2-negative human breast tumor samples showed the highest expression of *HIF-1α.* We also recorded that stage II and stage III tumors showed the highest expression of *HIF-1α*, indicating hypoxia enhances miRNA-induced aggressive breast cancer phenotypes at progressive disease states. In this same set of tumors, we previously published that miR526b and miR655 expression was high in tumors and high expression of both miRNAs were associated with poor-patient survival [13,14]. In tumor tissues, we also recorded a strong correlation between miR526b and *HIF-1α* and between miR655 and *HIF-1α*, which suggests that *HIF-1α* and miRNAs strongly interact to enhance breast cancer progression. These in situ data further confirmed the aggressive breast cancer phenotypes recorded in ER/PR-positive, HER2-negative, and miRNA-overexpressing MCF7-miR526b and MCF7-miR655 cell lines under hypoxia in the present article. To test the correlation between miRNA-cluster expressions with *HIF-1α* expression within tumors in an independent data set, we also extracted data from cBioPortal, which includes data from 16 different breast cancer studies. These independent data sets results also showed a strong and positive correlation between miR526b and miR655 cluster with HIF-1α expression, further strengthening our findings. Here, we discovered a novel collaboration between hypoxia and miR526b/miR655 in breast cancer metastasis. It would be interesting to investigate in the future if these two miRNAs can serve as breast cancer biomarkers, specifically in ER-positive breast cancer, which is the most common type of breast cancer incidence in Canada. 

## 4. Materials and Methods 

**Figure 13:**
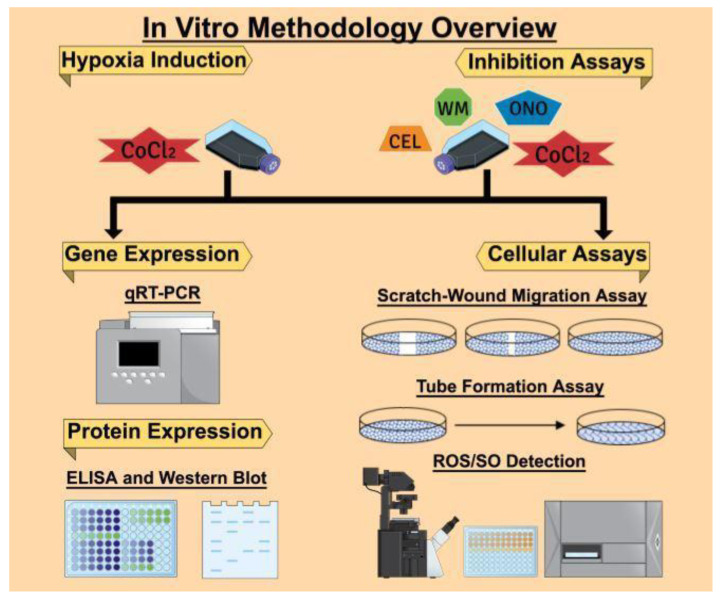
In vitro methodologies overview.

The overall in vitro methodologies followed in this article are presented in Figure 13. We used the Mind the Graph Platform to create the graphical images.

### 4.1. Ethics Statements

The experiments were conducted at the Department of Biology in Brandon University, following the regulations of Brandon University Research Ethics (#21986, approved on 21 April 2017) and Biohazard Committee (#2017-BIO-02, approved on 13 September 2017). 

### 4.2. Cell Culture

MCF7, T47D, SKBR3, Hs578T, and MDA-MB-231 breast cancer cell lines were purchased from the American Culture Type Collection (ATCC, Rockville, MD, USA). Stable miRNA-overexpression MCF7-miR526b, MCF7-miR655, and SKBR3-miR526b cell lines and COX-2-overexpressing MCF7-COX2 cell line were created by transfecting MCF7 and SKBR3 cells with respective miRNA or COX-2 overexpression plasmids. Transfected cell lines were sustained with Geneticin (Gibco, Mississauga, ON, Canada) following protocols as previously described [13,14,32]. MCF7, MCF7-miR526b, MCF7-miR655, and SKBR3-miR526b cell lines were grown in Roswell Park Memorial Institute (RPMI) 1640 medium (Gibco, Mississauga, ON, Canada), supplemented with 10% fetal bovine serum (FBS) and 1% Penstrep. T47D, SKBR3, MCF7-COX2, Hs578T, and MDA-MB-231 cell lines were grown in Dulbecco’s Modified Eagle Medium (DMEM) (Gibco, Mississauga, ON, Canada) supplemented with 10% FBS and 1% Penstrep. All cell lines were maintained in a humidified incubator at 37 °C and 5% CO_2_. MCF10A mammary epithelial cells were grown and maintained in Dr. Lala’s laboratory at the University of Western Ontario following ATCC protocol. An aliquot of cDNA samples was then transferred to Dr. Majumder laboratory at Brandon University.

### 4.3. Drugs and Chemicals

Celecoxib (COX-2 inhibitor, CEL) was purchased from Pfizer (Groton, CT, USA). ONO-AE3-208 (selective EP4 antagonist, EP4A, ONO) was a gift from ONO Pharmaceuticals (Osaka, Japan). Wortmannin (irreversible PI3K/Akt inhibitor, WM) was purchased from Sigma-Aldrich (Saint Louis, MO, USA). CoCl_2_ was purchased from Fisher Scientific (Mississauga, ON, Canada). For all treatments in vitro, hypoxia (CoCl_2_) served as the positive control and normoxia (sterile water, the solvent of CoCl_2_) served as the negative control. 

### 4.4. Hypoxia Induction In Vitro with CoCl_2_ Treatment

Concentrations of CoCl_2_ were chosen based on other publications tested with breast cancer cells [29,30]. Once 70% confluent, cells were serum starved for 12 h. and CoCl_2_ was administered at a concentration of either 50 µM or 150 µM. 24 h after CoCl_2_ treatment, cells were harvested for RNA extraction or used for cell functional assays as described below. We observed that 150 µM induced maximum HIF-1α expression (Figure S1), thus, for all treatments in vitro, 150 µM of CoCl_2_ treatment was considered as hypoxia, and sterile H_2_O served as normoxia.

### 4.5. RNA Extraction, cDNA Synthesis, and Quantitative Real-Time PCR 

Using the miRNeasy Mini Kit (Qiagen, Toronto, ON, Canada), total RNA extractions were done from non-treated T47D, SKBR3, MCF7-COX2, SKBR3-miR526b, Hs578T, MDA-MB-231, MCF7, MCF7-miR526b, and MCF7-miR655 cell lines, as well as CoCl_2_-treated MCF7, MCF7-miR526b, MCF7-miR655, SKBR3-miR526b, and MCF7-COX2 cell lines. The RNA was then reverse transcribed into cDNA using the High-Capacity cDNA Reverse Transcription Kit (Applied Biosystems, Waltham, MA, USA). 

For quantitative Real-Time PCR (qRT-PCR), the TaqMan Gene and miRNA Expression Assays were used. Two genes, *Beta-actin* (Hs01060665_g1) and *RPL5* (Hs03044958_g1) were used as endogenous control genes and RNU44 (assay ID #001094) was considered as an internal control miRNA. The expressions of pri-miR526b (Hs03296227_pri), pri-miR655 (Hs03304873_pri), hsa-miR-526b-5p (assay ID #002382), hsa-miR-655-3p (assay ID #001612), *HIF-1α* (Hs00153153_m1), *VEGFA* (Hs00900055_m1), *COX-2* (Hs00153133_m1), *EP4* (Hs00964382_g1), *VHL* (Hs03046964_s1), *NFκB1* (Hs00231653_m1), *TWIST1* (Hs04989912_s1), *VIM* (Hs00185584_m1), *SNAIL* (Hs00195591_m1), *CDH1* (Hs00170423_m1), *TXNRD1* (Hs00917067_m1), *SALL4* (Hs01010838_g1), *ZNF207* (Hs01045973_m1), and *NR2C2* (Hs00991824_m1) were quantified using relative gene expression analysis. A relative fold change of gene expression was used using the comparative threshold cycle (∆Ct) followed by fold change using the 2^-∆∆Ct^ method [3,13,14,32].

### 4.6. Enzyme-Linked Immunosorbent Assay (ELISA) Analysis of HIF-1α 

HIF-1α protein quantification was carried out using the ab171577-HIF1a Human SimpleStep ELISA Kit (Abcam, Toronto, ON, Canada). This assay is specific to the HIF-1α protein and does not cross-react with HIF-1α homologues, such as HIF-2α (EPAS-1). Three different passages of MCF7, MCF7-miR526b, MCF7-miR655, SKBR3-miR526b, and MCF7-COX2 cell lines were seeded into a six-well plate and grown to 80% confluence. Cells were treated with 150 µM CoCl_2_ for 24 h. Three experimental replicates were performed for each condition for each passage. The ELISA kit provided standards and was prepared following the manufacturer’s instructions. The cells were washed with PBS then solubilized with 1X Cell Extraction Buffer PTR. The cell lysate was then centrifuged, and the supernatant (total protein) was collected. In a 96-well plate, 50 μL of each of the sample protein and prepared standards were added to the wells. Additionally, 50 μL of the HIF-1α antibody cocktail was added, and the plate was incubated on a plate shaker. Next, the wells were washed with 1X Wash Buffer PT, and 100 µL of TMB Substrate was added to each well and incubated. Finally, 100 µL of Stop Solution was added to each well and mixed gently. Microplate readings were then recorded with OD at 450 nm to measure HIF-1α protein levels. Data was collected using the SoftMax Pro 6 Microplate Data Acquisition and Analysis software (Molecular Devices, San Jose, CA, USA). Calculations were performed following the manufacturer’s instructions. In all cases, negative control data were subtracted from experimental data for normalization. Provided samples were used to generate a standard curve for protein quantification.

### 4.7. Western Blot Analysis

Cells were treated with M-PER^®^ Mammalian Protein Extraction Reagent (Thermo Scientific, Rockford, IL, USA), HALT Protease Inhibitor Cocktail (Thermo Scientific), and Phosphatase Inhibitor Cocktail (Thermo Scientific) to extract total protein. Approximately, 15–20 µg of total protein were electrophoresed per well on an 8–10% SDS-polyacrylamide gel and transferred onto Immobilon-FL PVDF membranes (Millipore, Billerica, MA, USA). Blots were incubated with the HIF-1α primary antibody (H1alpha 67): sc-53546 (Santa Cruz Biotechnology, CA, USA) at 1:500 dilution and monoclonal GAPDH antibody (MAB374, from Millipore, Billerica, MA, USA) at 1:10,000 dilutions overnight. After blocking with primary antibodies, membranes were washed and then probed with a mixture of IRDye polyclonal secondary antibodies (LI-COR Biosciences, Lincoln, NE, USA). Images were read with an Odyssey infrared imaging system (LI-COR Biosciences, Lincoln, NE, USA).

### 4.8. Fluorescence Microplate Assay

Three different passages of MCF7, MCF7-miR526b, and MCF7-miR655 cell lines were seeded in a 96-well plate. Once the cells were grown to 70% confluency, they were treated with either 50 µM or 150 µM of CoCl_2_ for 24 h. ROS and SO levels were then detected using the ROS-ID Total ROS/SO detection kit (Enzo Life Sciences, Farmingdale, NY, USA), following the manufacturer’s protocol. First, the cells were washed with PBS to wash off cell culture media, and ROS/SO detection dyes were added to quantify ROS/SO production. One hour following the addition of detection dyes, microplate readings were done using the standard Fluorescein filter (Excitation/Emission: 485/535 nm) and Rhodamine filter (Excitation/Emission: 550/625 nm). Data was collected using the SoftMax Pro 6 Microplate Data Acquisition and Analysis software (Molecular Devices, San Jose, CA, USA). Concentrations of the ROS and SO produced by cells were determined based on the manufacturer’s instructions as we published earlier [27]. ROS/SO production was quantitatively shown as a ratio of hypoxia emissions (emissions from hypoxic cells) to negative control emissions (emissions from normoxic cells). The same process was used for the fluorescence microplate assay with the use of inhibitors, except cells were treated with 150 µM of CoCl_2_ and supplemented with either 20 µM Celecoxib, 50 µM ONO-AE3-208, or 10 µM Wortmannin for 24 h. To measure the effect of inhibitors, hypoxia treatment was considered as control. 

### 4.9. Fluorescence Microscopy Assay 

After total fluorescence emission measurement, we used the same ROS/SO detection kit to determine the total number of cells showing fluorescence and producing ROS and SO following the manufacturer’s protocol. MCF7, MCF7-miR526b, and MCF7-miR655 cell lines with or without CoCl_2_ were seeded in 96 well plates and grown until 70% confluent, then the cells were washed with PBS, and next, ROS/SO detection dyes were added. After 15 min of incubation, images were captured with a Nikon Ds-Ri1 microscopy camera and data were analyzed using the NIS Elements Advanced Research software (Nikon, Melville, NY, USA). The fluorescent cells in each experiment were quantified using the ImageJ software (National Institute of Health, Bethesda, MD, USA) as previously described [27]. For each condition, the negative control (normoxia) was used as a threshold for quantification of hypoxia effect. Total ROS/SO production was presented as quantification ratios, which were calculated by dividing all quantifications by negative control quantifications (i.e., the number of fluorescence-positive cells in the sample divided by the number of fluorescence-positive cells in the control), then dividing the resulting number by the total number of cells present in the well. The same process was used for the fluorescence microscopy assay with the use of inhibitors, except cells were treated with 150 µM of CoCl_2_ and supplemented with either 20 µM Celecoxib, 50 µM ONO-AE3-208, or 10 µM Wortmannin. To measure the effect of inhibitors, hypoxia treatment was considered as control.

### 4.10. Scratch-Wound Migration Assay

Cells were grown in RPMI complete (serum-supplemented) media until 90% confluent, then harvested and resuspended in complete RPMI, after which 300 μL of suspended cells (approximately 20,000 cells/mL) was added to a six-well cell culture plate and maintained until 90% confluency. A sterile 2 μL pipette tip was used to scratch the surface of each well, after which the cells were washed with PBS to remove detached cells. The treatment conditions were then applied to the wells. For the hypoxia-mediated migration assay, sterile H_2_O was used as the negative control (normoxia), and 150 μM of CoCl_2_ treatment was considered as hypoxia. A total of 2 mL of the respective conditions (treatments in RPMI basal media) were added to each well. The migratory progress and wound size were captured using a Nikon Ds-Ri1 microscope camera at 0, 16, 24, and 48 h time points. To ensure that we were taking pictures of the same wound-healing site over time, each well was separated into four quadrants manually with a marker pen, and a wound/scratch was made once per coordinate. Additionally, the microscope’s coordinate system was used for double validation to ensure photos were taken in the same field of view. We have captured five pictures per quadrant to ensure that the entire wound was captured. Thus, per well, we took at least 20 pictures. NIH ImageJ software was used to measure the width of the scratch wound in pixels and mean data of 20 pictures were considered as data for a single experimental replicate. We used three experimental wells or replicates and three biological replicates per condition [3]. The same process was used for the migration assay with the use of inhibitors, to determine the roles of COX-2, EP4 receptor, and the PI3K/Akt signaling pathways, except the cells were seeded in 24-well plates and treated with 150 μM CoCl_2_ for 24 h, then supplemented with either 20 µM Celecoxib, 50 µM ONO-AE3-208, or 10 µM Wortmannin for another 24 h. All quantifications were done after 24 h of inhibitor treatment (which is equivalent to 48 h of CoCl_2_ treatment), as we found an increase in cell death and difficulty in quantification after 24 h.

### 4.11. Tube Formation Assay

Tube formation assays were carried out as previously described in a 24-well plate [3]. Diluted Matrigel media was prepared as a 1:1 ratio of growth factor reduced Matrigel (BD Biosciences, Bedford, Massachusetts, USA) and basal RPMI media. 200 µL of diluted Matrigel was added to each well in a 24-well plate and incubated for 1 h at 37 °C to allow the Matrigel to crosslink and form the extracellular matrix. Next, 200 µL cells were then seeded into the Matrigel coated plate with a density of approximately 20,000 cells per well. MCF7, MCF7-miR655 and MCF7-miR526b cell lines were resuspended in either RPMI complete media; CoCl_2_ (150 µM)-RPMI media; or CoCl_2_-RPMI media along with either 20 µM Celecoxib (COX-2 inhibitor) or 50 µM ONO-AE3-208 (EP4 antagonist), or 10 µM Wortmannin (Irreversible PI3K/Akt pathway inhibitor) as inhibitory conditions. Each condition was tested twice (experimental replicates) and repeated three times (biological replicates). Tube formation was measured at 24 and 48 h, and images were obtained using a Nikon inverted microscope. Quantification of tubes and branching points was carried out using NIH ImageJ software (NIH, Bethesda, MD, USA). 

### 4.12. Bioinformatics Analysis

miRbase [36] and Enrichr [57] were two online databases used for conducting bioinformatics analysis in this study. miRbase is a miRNA database, which provides predicted miRNA target genes along with miRNA cluster information. The complete target gene list for miR526b and miR655 was downloaded using TargerScanVert release 7.1 [58] in miRbase for five prime mature sequences hsa-mir-526b and hsa-mir-655. The Enrichr database uses enrichment analysis to identify transcription factors regulating genes. All transcription factors associated with *PTEN* and *NFκB1* were downloaded. The two lists generated from miRbase and the Enrichr database were then cross-examined to determine shared target genes and transcription factors.

### 4.13. Human Breast Cancer Tissue Samples

Frozen human breast tissue samples were obtained from the Ontario Tumour Bank after ethical approval by Ontario Cancer Research Ethics Board (Tec # 010-11), then following approval by the Ethics Review Board of the Tumor bank and collected at the University of Western Ontario at Dr. Lala’s laboratory. Qiagen miRNeasy mini kit was used to extract mRNA or miRNA from tissue samples, followed by cDNA synthesis using cDNA Reverse Transcription Kit (Applied Biosystems, USA). An aliquot of all the cDNA samples were transferred to Dr. Majumder’s laboratory at Brandon University following the Material Transfer Agreement (MTA) between Brandon University and the University of Western Ontario. All further experiments were conducted at the Department of Biology at Brandon University following Brandon University Ethics and Biohazard protocols. 

### 4.14. In Silico Analysis of cBioPortal Data via TCGA

miR526b and miR655 cluster information was extracted from the miRbase miRNA database [36]. We identified that there are 20 miRNAs within each miRNA cluster for miR526b and miR655. Next, we used the cBioPortal database within TCGA, which includes data from 16 breast cancer studies to extract miR526b and miR655 cluster miRNA expression, along with *HIF-1α* mRNA expression, which were both presented as z-scores [37,38]. For the miR526b miRNA cluster, the cBioPortal database contained miRNA expression data for miR516a-1 and miR516a-2. As for miR655s miRNA cluster, nine miRNA had expression in the cBioPortal database (miR154, miR369, miR381, miR382, miR409, miR410, miR487b, miR539, and miR889). The mean of available miRNAs z-score within each cluster was considered and compared to the *HIF-1α* z-score to determine a correlation between miR526b and miR655 miRNA clusters and *HIF-1α*.

### 4.15. Statistical Analysis

Statistical calculations were performed using GraphPad Prism (GraphPad Software, Version 8.4.3., San Diego, CA, USA). All parametric data were analyzed with one-way analysis of variance (ANOVA) by Tukey–Kramer or Dunnett post-hoc comparisons. The student’s t-test was used when comparing the means of two datasets, and Pearson’s correlation coefficient was employed to assess statistical correlations. Statistically relevant differences between means were accepted at *p* < 0.05. 

## 5. Conclusions

Although the roles of miR526b, miR655, and hypoxia are independently studied in various tumor metastasis models, this is the first time an association between miR526b, miR655, and hypoxia to promote metastatic breast cancer phenotypes has been established. These findings further strengthen the roles of these two miRNAs as master regulators of the tumor microenvironment in promoting breast cancer. In addition, these miRNAs can serve as possible therapeutic targets in ER/PR-positive and HER2-negative miR526b/miR655-high breast cancer. 

## Figures and Tables

**Table 1:** Demography of human benign and malignant tissue samples from Ontario Tumor Bank: this table illustrates tobacco exposures, alcohol consumption, hormone receptor status (ER, PR, HER2), and tumor stage status of the benign and malignant human tissue samples used in this study. Samples were age-matched; the majority of samples are from female patients, only three samples are male. Hormone receptor status of weak and intermediate was considered neither negative nor positive. Age and pack year were presented as mean ± SD.

Subjects		Benign *N* = 20 (%)	Malignant *N* = 96 (%)
Sex	Female	20 (100)	93 (96.88)
	Male	0 (0)	3 (3.13)
Age Distribution (Years)	Range	52–87	27–92
Age (years)	Mean ± SD	66 ± 11	63 ± 17
Smoking	Smokers	1 (5)	24 (25)
	Pack Year (PY) ± SD	40	27 ± 19
Alcohol Consumption	Social or Occasional Drinker	5 (25)	28 (29.17)
	Regular Drinker	0 (0)	3 (3.13)
ER Status	Positive	N/A	37 (38.58)
	Negative	N/A	19 (19.79)
	Unknown	N/A	6 (6.25)
PR Status	Positive	N/A	31 (32.29)
	Negative	N/A	30 (31.25)
	Unknown	N/A	6 (6.25)
HER2 Status	Positive	N/A	21 (21.88)
	Negative	N/A	61 (63.54)
	Unknown	N/A	14 (14.58)
ER, PR, HER2 Status	Negative	N/A	10 (10.42)
Tumor Stage			
I *		N/A	7 (7.29)
II		N/A	45 (46.87)
III		N/A	39 (40.63)
IV		N/A	5 (5.21)

N/A: Not Applicable. * Stage 0 samples (*n* = 2) were compiled with stage I samples and considered as stage I (*n* = 5).

## Supplementary Materials

The following are available online at https://www.mdpi.com/2072-6694/12/8/2008/s1, Figure S1: CoCl_2_ dose-response assay; Figure S2: miRNA, pri-miRNA and COX-2 expression in various cell lines; Figure S3: Western blot analysis to measure endogenous HIF-1α expression in various breast cancer cell lines; Figure S4: MCF7-miR526b and MCF7-miR655 cell densities increase due to hypoxia; Figure S5: MCF7-miR655 cell migration in normoxia and hypoxia; Figure S6: Hypoxia-enhanced vascular mimicry and inhibition of tube formation with COX-2-I, EP4A and PI3K/Akt-I; Figure S7: Inhibition of hypoxia-enhanced migration for MCF7-miR655 cells

## Abbreviations

CEL	Celecoxib
CDH1	E-cadherin (Epithelial cadherin)
CoCl_2_	Cobalt chloride
COX-2	Cyclooxygenase-2
CPEB2	Cytoplasmic polyadenylation element-binding protein 2
ELISA	Enzyme-linked immunosorbent assay
EMT	Epithelial-to-mesenchymal transition
EP4	Prostaglandin E_2_ receptor 4
ER	Estrogen receptor
HER2	Human epidermal growth factor receptor 2
HIF-1α	Hypoxia-inducible factor 1-alpha
miRNA/miR	MicroRNA
mRNA	Messenger RNA
NFκB	Nuclear Factor Kappa-light-chain-enhancer of activated B cells
NFκB1	Nuclear factor NF-kappa-B p105 subunit
NR2C2	Testicular receptor 4
ONO	ONO-AE3-208
PGE2	Prostaglandin E₂
PHD	Prolyl hydroxylase domain
PI3K	Phosphoinositide 3-kinases
PR	Progesterone receptor
Pri-miRNA	Primary miRNA
PTEN	Phosphatase and tensin homolog
ROS	Reactive oxygen species
SALL2	Sal-like protein 2
SALL4	Sal-like protein 4
SLC	Stem-like cell
SNAIL	Zinc finger protein SNAI1
SO	Superoxide
TCGA	The Cancer Genome Atlas
TWIST1	TWIST1-related protein 1
TXNRD1	Thioredoxin reductase 1
VEGF	Vascular endothelial growth factor
VEGFA	Vascular endothelial growth factor A
VHL	Von Hippel–Lindau tumor suppressor
VIM	Vimentin
WM	Wortmannin
ZNF207	Zinc finger protein 207
