# Supplementary material for: Chemically Induced Hypoxia Enhances miRNA Functions in Breast Cancer
Source: Cancers (Basel). 2020 Jul 22;12(8):2008. doi: 10.3390/cancers12082008 (PMC7465874; doi:10.3390/cancers12082008)
Supplement: Supplementary file 1 [file cancers-12-02008-s001.pdf]

cancers

Supplementary Materials

Chemically Induced Hypoxia Enhances miRNA Functions in Breast Cancer

Emma Gervin, Bonita Shin, Reid Opperman, Mackenzie Cullen, Riley Feser, Sujit Maiti and Mousumi Majumder

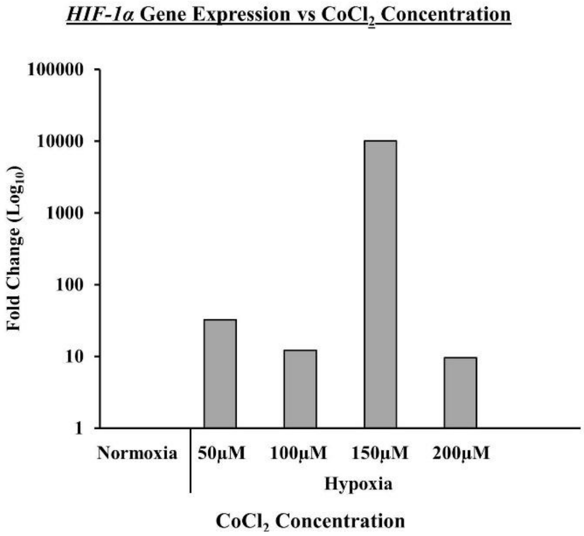

Figure S1. $CoCl_2$ dose-response assay: Dose-response graph of hypoxic marker *HIF-1α* gene expression with various $CoCl_2$ concentrations in MCF7-miR526b cells. Cells were treated for 24 h.

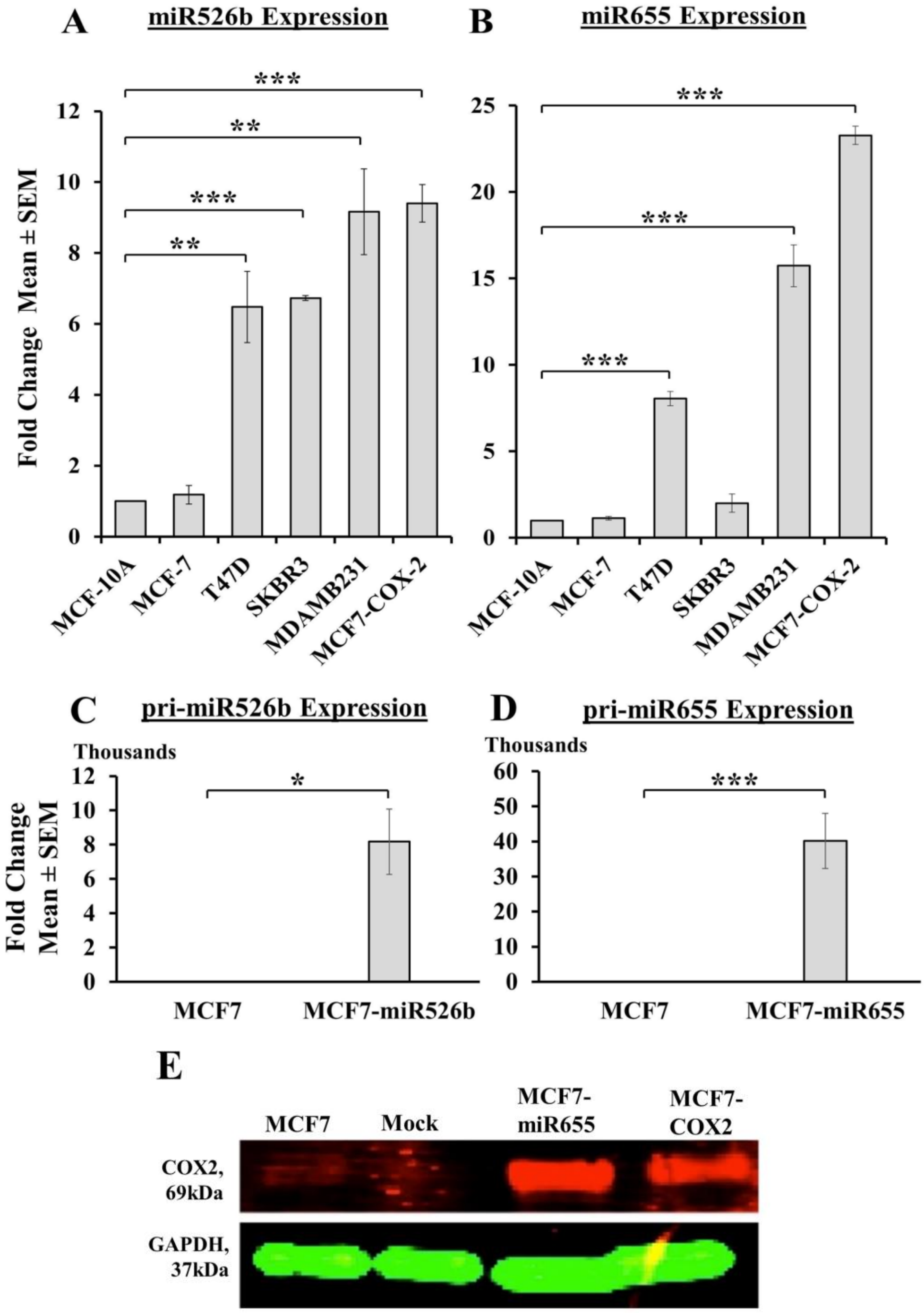

Figure S2. miRNA, pri-miRNA and COX-2 expression in various cell lines: (**A**) miR526b expression in various breast cancer cell lines. (**B**) miR526b expression in various breast cancer cell lines. (**C**) Pri-miR526b expression in MCF7 and MCF7-miR526b cell lines. (**D**) Pri-miR655 expression in MCF7 and MCF7-miR526b cell lines. (**E**) Western blots analysis of COX-2 protein expression in MCF7, MCF7-Mock, MCF7-miR655 and MCF7-COX2 cell lines. Data in **A** was previously published in [1], data in **B** and **E** was previously published in [2]. * $p < 0.05$, ** $p < 0.001$ and *** $p < 0.0001$.

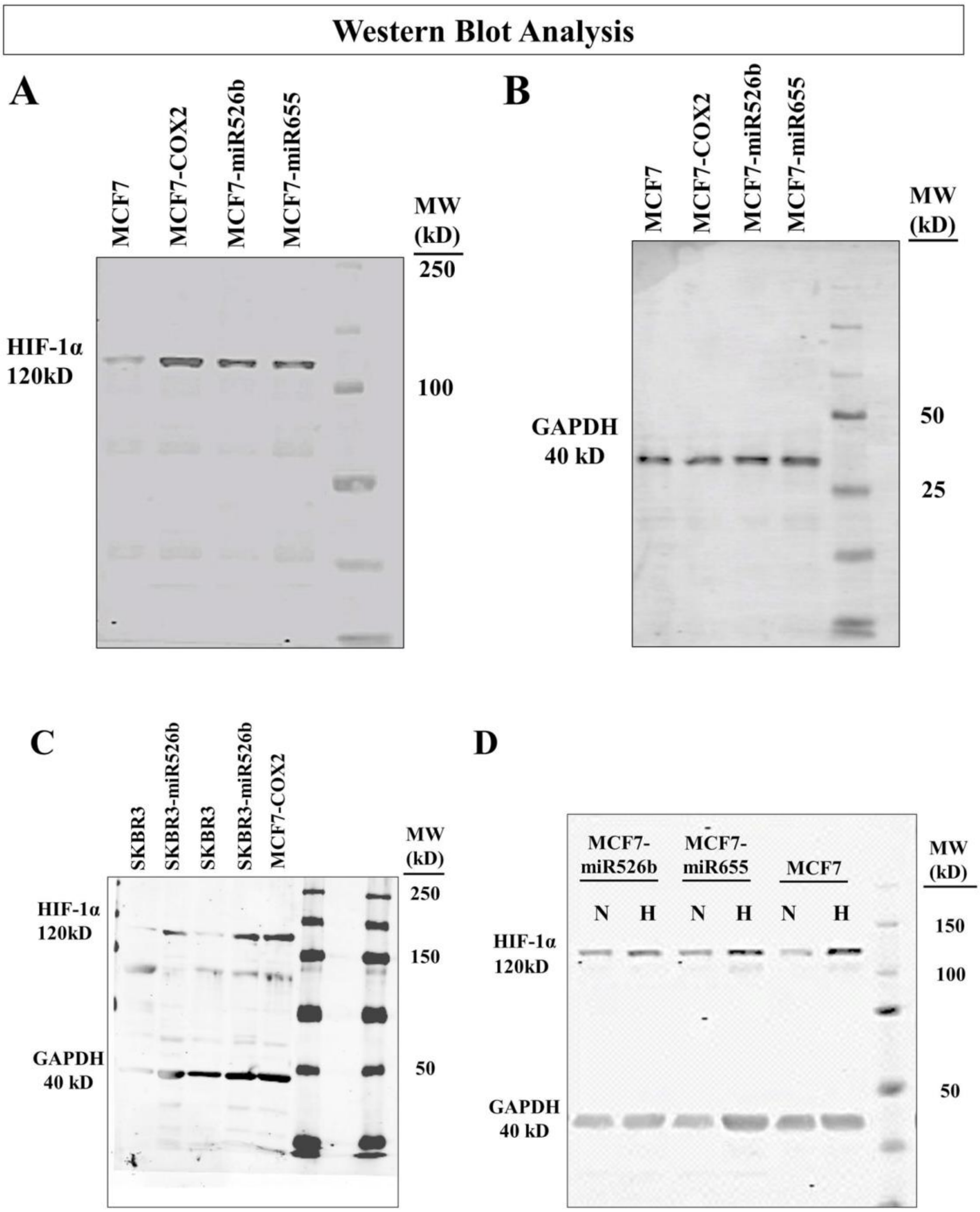

Figure S3. Western blot analysis to measure endogenous HIF-1α expression in various breast cancer cell lines: (**A**) Endogenous total HIF-1α protein expression in MCF7, MCF7-miR526b, MCF7-miR655, MCF7-COX-2 cell lines and (**B**) Corresponding cell lines GAPDH protein expression (control). (**C**) Endogenous total HIF-1α and GAPDH protein expression in SKBR3 cell lines and MCF7-COX2 (positive control). (**D**) HIF-1 protein expression in MCF7, MCF7-miR526b and MCF7-miR655 cell lines in normoxia and hypoxia, GAPDH served as control protein.

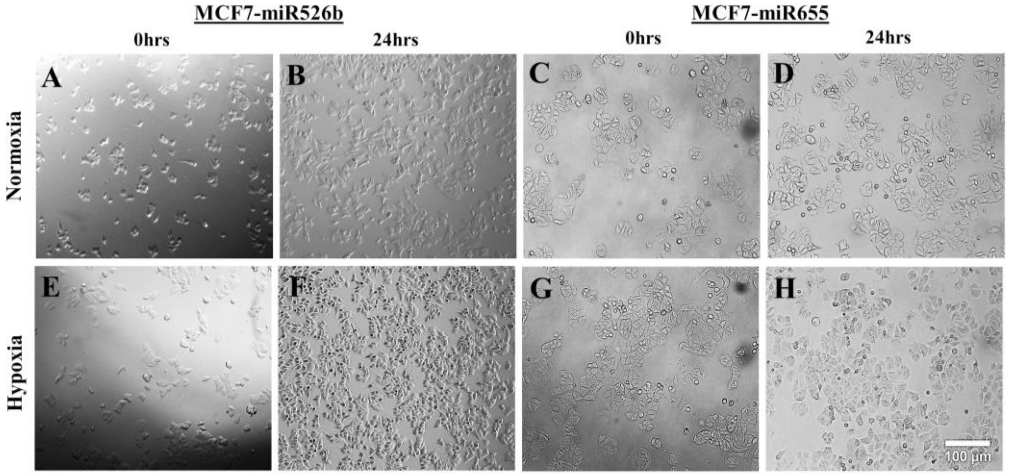

Figure S4. MCF7-miR526b and MCF7-miR655 cell densities increase due to hypoxia: (**A**–**D**) Cell densities of MCF7-miR526b and MCF7-miR655 cells in normoxia at 0 and 24 h. (**E**–**H**) Cell densities of MCF7-miR526b and MCF7-miR655 cells in hypoxia at 0 and 24 h (150 μM $CoCl_2$).

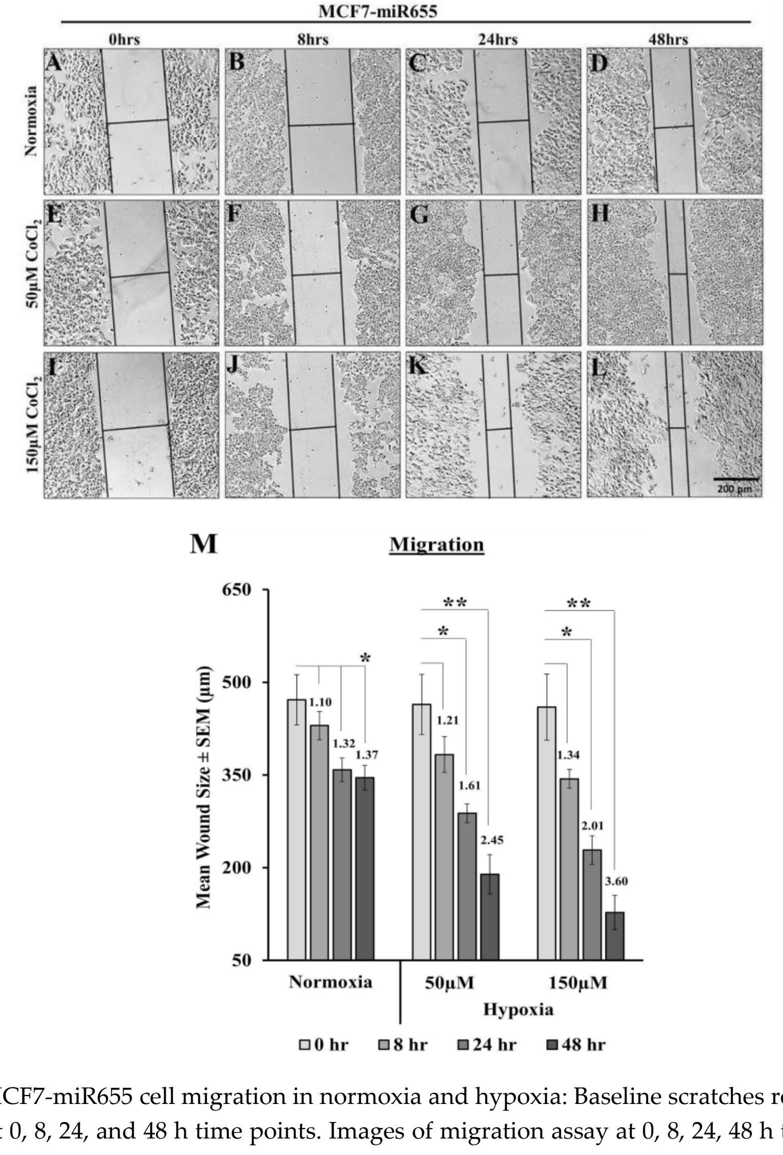

Figure S5. MCF7-miR655 cell migration in normoxia and hypoxia: Baseline scratches represented by black lines at 0, 8, 24, and 48 h time points. Images of migration assay at 0, 8, 24, 48 h time points in (**A**–**D**) normoxia, (**E**–**H**) 50 μM $CoCl_2$ treatment and (**I**–**L**) 150 μM $CoCl_2$ treatment. Scale bar represents 200 μM. (**M**) Quantitative wound size measurement at 0, 8, 24, and 48 h time points. Data presented as the mean migration distance ± SEM of triplicate replicates; * $p < 0.05$ and ** $p < 0.01$.

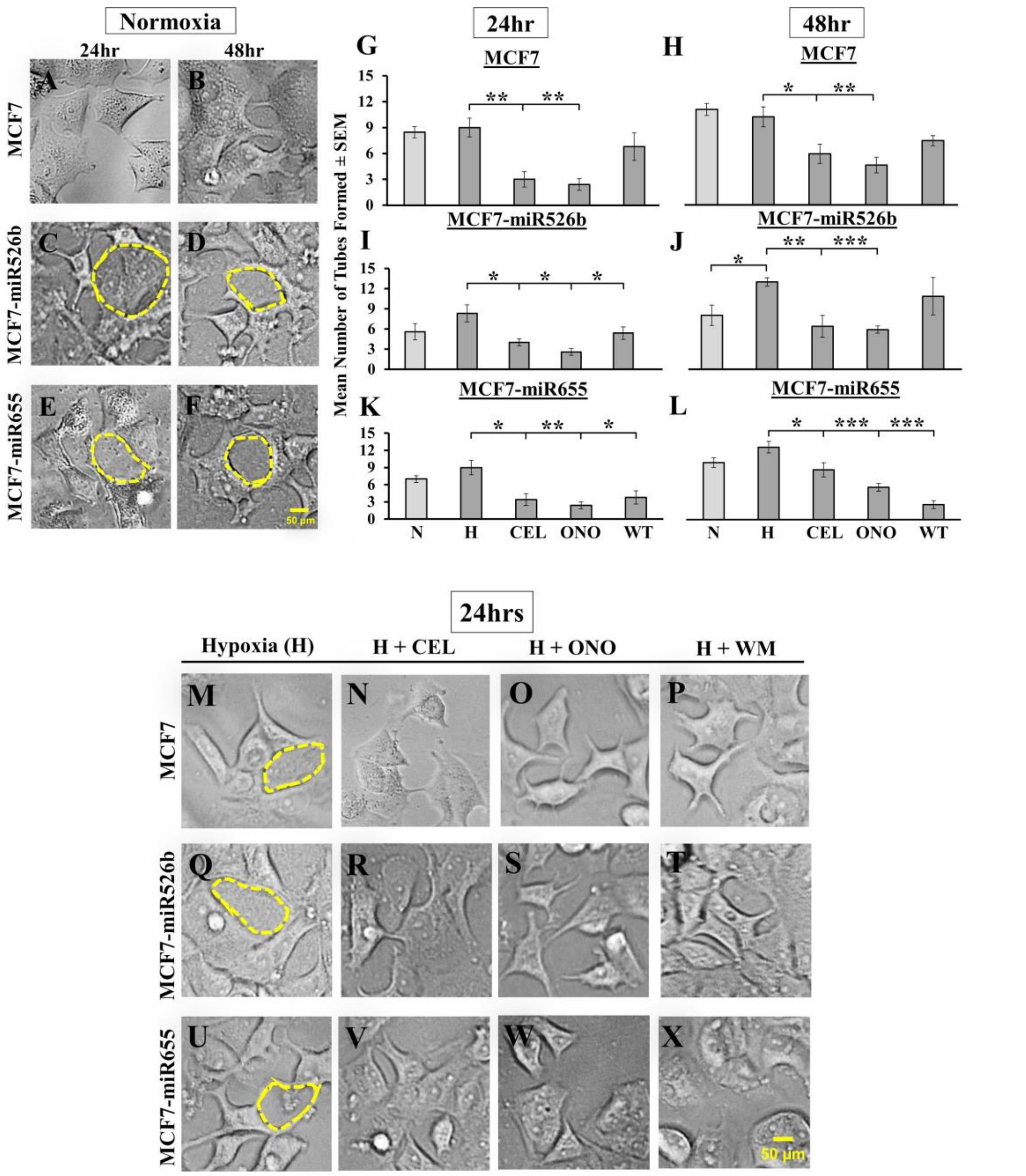

Figure S6. Hypoxia-enhanced vascular mimicry and inhibition of tube formation with COX-2-I, EP4A and PI3K/Akt-I: Tubes are outlined as yellow dotted lines. Normoxic conditions for MCF7, MCF7-miR526b and MCF7-miR655 cells respectively at (**A**, **C**, **E**) 24 and (**B**, **D**, **F**) 48 h. Quantitative data for MCF7, MCF7-miR526b and MCF7-miR655 cells respectively at (**G**, **I**, **K**) 24 and (**H**, **J**, **L**) 48 h. Hypoxic conditions at 24 h for MCF7, MCF7-miR526b and MCF7-miR655 cells respectively in (**M**, **Q**, **U**) hypoxia, (**N**, **R**, **V**) hypoxia with CEL, (**O**, **S**, **W**) hypoxia with ONO, and (**P**, **T**, **X**) hypoxia with WM. Data presented as the mean migration distance ± SEM of triplicate replicates; * $p < 0.05$, ** $p < 0.01$ and *** $p < 0.001$.

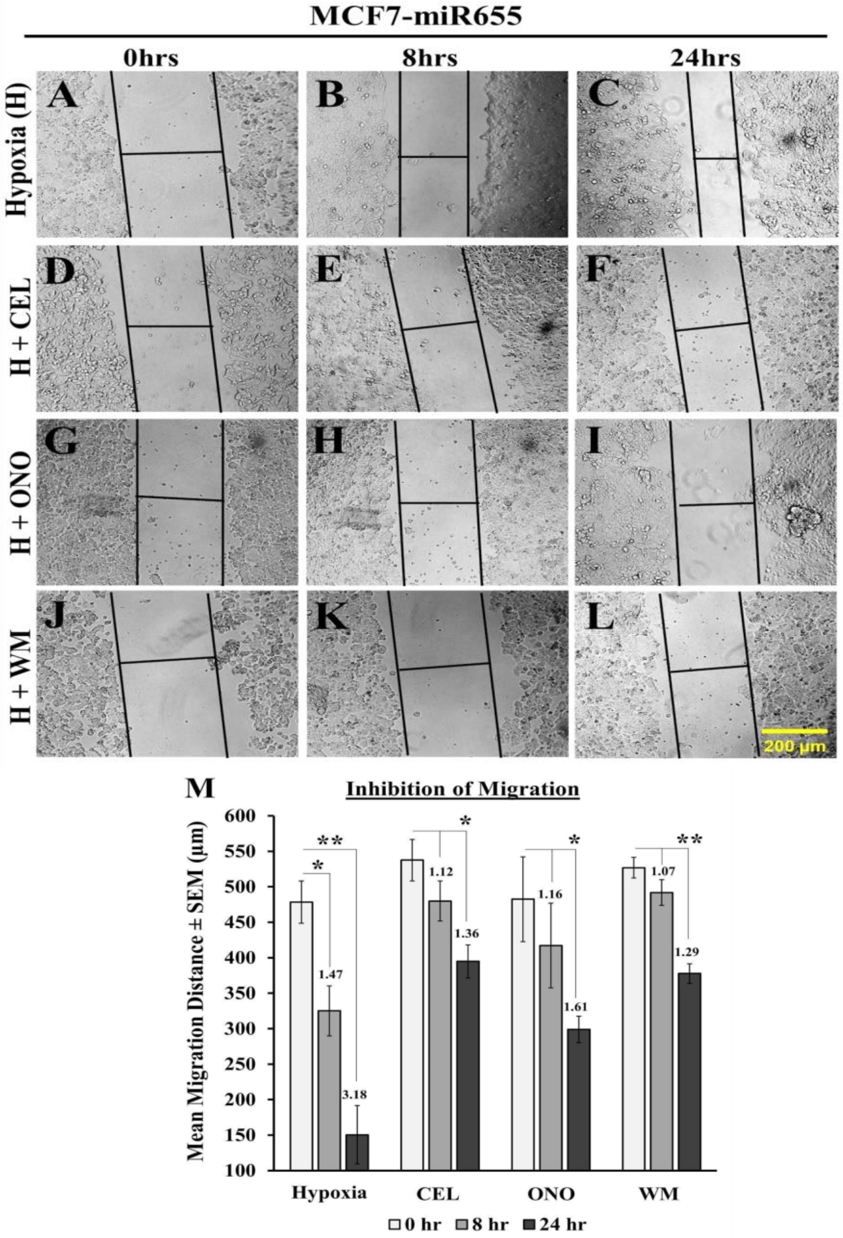

Figure S7. Inhibition of hypoxia-enhanced migration for MCF7-miR655 cells: Baseline scratches represented by black lines at 0, 8, 24, and 48 h. time points. Images of MCF7-miR655 at 0, 8, 24, 48 h. in (**A**–**C**) hypoxia, (**D**–**F**) hypoxia with CEL, (**G**–**I**) hypoxia with ONO, (**J**–**L**) hypoxia with WM. Scale bar represents 200 µM. (**M**) Quantitative analysis of wound size. Data presented as the mean migration distance ± SEM of triplicate replicates; * $p < 0.05$ and ** $p < 0.01$.

References

1. Majumder, M.; Landman, E.; Liu, L.; Hess, D.; Lala, P.K. COX-2 Elevates Oncogenic miR-526b in Breast Cancer by EP4 Activation. *Mol Cancer Res* **2015**, *13*, 1022-1033, doi: 10.1158/1541-7786.MCR-14-0543.
2. Majumder, M.; Dunn, L.; Liu, L.; Hasan, A.; Vincent, K.; Brackstone, M.; Hess, D.; Lala, P.K. COX-2 induces oncogenic micro RNA miR655 in human breast cancer. *Sci. Rep* **2018**, *8*, 327, doi: 10.1038/s41598-017-18612-3.

© 2020 by the authors. Licensee MDPI, Basel, Switzerland. This article is an open access article distributed under the terms and conditions of the Creative Commons Attribution (CC BY) license (http://creativecommons.org/licenses/by/4.0/).
